# Seed priming with silicon quantum dots promotes maize seedling establishment in coastal saline soil

**DOI:** 10.3389/fpls.2026.1796045

**Published:** 2026-04-14

**Authors:** Lize Zhao, Tianwen Wang, Qiang Liu, Kun Chen, Peitong Li, Shoushuo Zhang, Hao Zheng, Fengmin Li, Xianxiang Luo

**Affiliations:** 1 Institute of Coastal Environmental Pollution Control, Key Laboratory of Marine Environment and Ecology, Frontiers Science Center for Deep Ocean Multispheres and Earth System, Ocean University of China, Qingdao, China; 2 Laboratory for Marine Ecology and Environmental Science, Qingdao Marine Science and Technology Center, Qingdao, China

**Keywords:** physiological evidence, salt stress, seed priming, silicon quantum dots, yellow river delta

## Abstract

**Introduction:**

Developing efficient, eco-friendly strategies to enhance crop salt tolerance is essential for sustaining grain productivity in saline soils. Although silicon quantum dots (Si QDs) have been reported to enhance crop stress resilience through soil or foliar application, their potential as seed priming agents to regulate crop salt tolerance remains poorly understood.

**Methods:**

To address this question, we synthesized water-soluble Si QDs via a one-step hydrothermal method. Using maize (*Zea mays* L.) as the model crop, we first performed plate germination assays to compare Si QDs priming effects with those of hydropriming, sodium silicate (Na_2_SiO_3_), and silica nanoparticles (SiO_2_ NPs). We further conducted pot experiments to systematically evaluate the growth-promoting efficacy and physiological basis of Si QDs seed priming in Yellow River Delta coastal saline soil.

**Results and Discussion:**

At high salinity (125–150 mM), Si QDs seed priming significantly enhanced the germination rate, germination potential, germination index, root length, shoot length, and vigor index in maize. Si QDs seed priming improved the overall germination performance by 132.3% and 39.9% relative to Na2SiO3 and SiO2 NPs priming, respectively. Pot experiments demonstrated that Si QDs seed priming enhanced seedling emergence, biomass accumulation, and root system architecture. Notably, Si QDs seed priming significantly improved seedling responses to salt stress, as reflected by a 19.5% decrease in the Na^+^/K^+^ ratio, a 10.1% increase in superoxide dismutase activity, a 25.4% decrease in malondialdehyde content, and a marked enhancement in photosynthetic capacity. With ultrasmall particle size and abundant hydrophilic functional groups, Si QDs form effective interfacial contact with the seed coat, and are efficiently internalized by seed tissues, thereby enhancing germination. In addition, Si QDs promote maize seedling growth via synergistic regulation of ion homeostasis, enhancement of antioxidant defense, and improvement of photosynthetic efficiency. Collectively, this study not only demonstrates that Si QDs outperform conventional Na2SiO3 and SiO2 NPs as seed priming agents, but also expands the application scope of Si QDs from post-emergence field application to pre-sowing seed priming. These findings provide experimental evidence supporting potential application of Si QDs in Yellow River Delta coastal saline soil.

## 1 Introduction

Amid the ongoing shrinkage of global arable land and escalating global food security risks, reclamation of 1.0 × 10^9^ ha of salt-affected soils is imperative for expanding agricultural frontiers (Wang et al., 2023; Chen et al., 2024). However, staple cereal crops, such as maize, wheat (*Triticum aestivum* L.), and rice (*Oryza sativa* L.), which account for the majority of human caloric intake, are highly sensitive to salt stress (Rawat et al., 2022). Excessive Na^+^ accumulation primarily induces osmotic stress, leading to cellular dehydration, diminished stomatal conductance, and impaired photosynthetic performance (Sun et al., 2025). Furthermore, competitive Na^+^ influx suppresses the uptake of essential macronutrients, including K^+^ and Ca^2+^, and triggers the overproduction of reactive oxygen species (ROS). This ionic imbalance, coupled with the subsequent oxidative burst, causes severe oxidative damage and compromises the integrity of cellular membranes (Hassani et al., 2021). Collectively, these effects manifest as pronounced growth inhibition, reduced biomass accumulation, compromised crop quality, and ultimately, plant mortality. Serving as the core zone for the national strategy of the Ecological Protection and High-quality Development of the Yellow River Basin (Luo et al., 2024), the Yellow River Delta represents a typical coastal salt-affected soil ecosystem in northern China (Zhang et al., 2023). Encompassing approximately 2.051×10^5^ ha of salinized land (Li et al., 2023a), this region holds substantial potential for agricultural production expansion (Chen et al., 2022). Factors including flat topography, shallow groundwater tables, poor drainage, and seawater intrusion have collectively driven widespread salinization of surface soils (Chen et al., 2019). Furthermore, during arid seasons with intense evaporation, capillary rise and subsequent solute enrichment drive the upward migration of salts, leading to localized topsoil resalinization, which severely constrains local agricultural productivity (Fu et al., 2020; Bimurzayev et al., 2021). The strategy of adapting crops to saline conditions has attracted increasing attention due to its cost-effectiveness and sustainability (Wang et al., 2025). For instance, while hybrid breeding and gene editing technologies hold great promise for enhancing crop stress tolerance, their widespread field application is frequently constrained by long development cycles and unpredictable agronomic performance (Liu et al., 2024a). Therefore, developing highly efficient, economically viable, and ecologically safe interventions to enhance crop salt tolerance is of paramount importance for unlocking the agricultural potential of salt-affected soils and safeguarding global food security.

The development of nanotechnology has provided new opportunities for agricultural development and sustainable transformation (Wu et al., 2025). Among these advances, seed nanopriming has emerged as a highly successful practice, significantly enhancing seed germination rates and seedling establishment quality (Wu and Li, 2022). The advantage of this technique lies in its exceptional scalability: an ultralow dosage of nanoparticles (NPs) is sufficient to treat a substantial volume of seeds, thereby conferring cost-effectiveness compared to conventional foliar application or soil-based application. Furthermore, residual NPs adhered to the seed surface after priming can be effectively removed through simple aqueous rinsing, thereby greatly reducing the potential ecological and human health risks associated with nanomaterial application (Zhao et al., 2024). Mechanistically, NPs interact with the seed coat and internal seed tissues through passive diffusion, endocytosis, and transmembrane transport proteins. These internalization pathways facilitate water uptake, storage reserve mobilization, and early metabolic activation. Concurrently, NPs modulate redox homeostasis by regulating ROS balance and antioxidant defense networks, and trigger the expression of stress-responsive defense genes, ultimately enhancing germination efficiency and conferring post-germination abiotic stress tolerance in seedlings (Ulhassan et al., 2023; Salam et al., 2025; Ulhassan et al., 2025). For example, seed priming with silver nanoparticles (Ag NPs) in maize activates plant hormone signal transduction and mitogen-activated protein kinase (MAPK) signaling cascades, conferring enhanced resilience against combined abiotic stresses (e.g., concurrent drought–cold and drought–salinity). Furthermore, the stress memory induced by Ag NPs can persist throughout the subsequent developmental stages (Chen et al., 2023). Despite these promising results, the widespread agricultural application of engineered metal-based nanomaterials is frequently constrained by their suboptimal biocompatibility and potential ecotoxicological risks (Lyu et al., 2025; Xiao et al., 2025). In contrast, non-metallic and biopolymer-based NPs, such as chitosan nanocarriers, silicon nanoparticles (Si NPs), and selenium nanoparticles, generally exhibit superior biocompatibility and therefore hold greater promise as nanomaterials for agricultural applications (Javaid et al., 2024; Xing et al., 2024; Zhuang et al., 2025).

Silicon (Si), the second most abundant element in the Earth’s crust after oxygen, is ubiquitously distributed throughout terrestrial soil and plant systems. Si NPs exhibit exceptional biocompatibility and can undergo biotransformation into silicic acid within the tissues of crops such as rice, wheat, and maize (Park et al., 2009). The subsequent biosilicification process enhances cell wall rigidity, thereby mitigating both biotic (e.g., pathogen infection) and abiotic (e.g., drought and salinity) stresses, and ultimately promoting plant growth (Khan, 2025). Seed priming with Si NPs improves germination efficiency and crop stress adaptability by regulating metabolic and transcriptional reprogramming in seeds (Rakesh et al., 2024). For instance, priming cucumber (*Cucumis sativus* L.) seeds with SiO_2_ NPs enhanced drought tolerance in both seeds and seedlings. This effect was achieved by elevating the levels of key metabolites, including sugars, amino acids, and antioxidants, and by modulating the expression of drought-responsive genes, which ultimately led to improved cucumber yield and nutritional quality (Zhuang et al., 2025). Similarly, SiO_2_ NPs priming in maize enhanced the accumulation of osmotic regulators such as soluble sugars, increased antioxidant enzyme activities, and maintained Na^+^/K^+^ homeostasis, thereby conferring pronounced salt tolerance (Alsamadany et al., 2024). Critically, under severe salt stress, osmotic restrictions and specific ion toxicities synergistically inhibit seed imbibition and hypocotyl elongation, indicating that material properties such as particle size, aqueous dispersibility, and cross-tissue translocation efficiency may be critical determinants of priming efficacy (Khan et al., 2023; Soni et al., 2026). Compared to conventional silicon-based nanoparticles, Si QDs represent a novel class of nanomaterials characterized by their ultrasmall particle size, superior aqueous dispersibility, enhanced biocompatibility, and exceptional photoluminescent properties. These attributes facilitate highly efficient tissue internalization and the subsequent modulation of plant physiological processes (Li et al., 2019, 2020). For example, soil application of Si QDs significantly amplified the light-harvesting capacity of lettuce (*Lactuca sativa* L. var. *ramose* Hort.), upregulated aquaporin gene expression, and promoted plant growth (Li et al., 2020b). In soil-grown maize, Si QDs upregulated the expression of biosynthetic genes responsible for structural and biochemical defenses, including those involved in lignin and benzoxazinoid production. This effect conferred enhanced resistance against the oriental armyworm (*Mythimna separata*, Walker) and contributed to increased grain yield (Xiao et al., 2023). Similarly, foliar application of Si QDs in rice enhanced nutrient uptake and upregulated genes associated with the metabolism of antioxidant compounds such as oxalic acid, maleic acid, and glycine, culminating in enhanced biomass accumulation (Jiang et al., 2024). Collectively, these studies underscore the considerable application potential of Si QDs for enhancing plant stress tolerance and growth. However, current research on the agricultural application of Si QDs has predominantly focused on soil and foliar application (Meng et al., 2026); their efficacy and potential advantages as seed priming agents, particularly within the context of the coastal saline soil in the Yellow River Delta, remain to be systematically elucidated.

Against this background, the scientific question of this study was to determine whether Si QDs exhibit higher efficacy compared to soluble silicate (Na_2_SiO_3_) and silica nanoparticles (SiO_2_ NPs) as seed priming agents for maize to improve germination efficiency under salt stress, and whether this advantageous effect is sustained from the germination stage through to seedling establishment in saline soil. We hypothesized that Si QDs could promote maize seed germination and seedling establishment under salt stress, and that this beneficial effect is primarily attributed to their ultrasmall particle size, favorable aqueous dispersibility, and abundant hydrophilic surface functional groups. Owing to these unique physicochemical properties, Si QDs can effectively interact with the seed coat and penetrate into seed and seedling tissues, thereby enhancing seedling salt tolerance by improving ion homeostasis, strengthening antioxidant defense, and preserving photosynthetic function. To verify these hypotheses, Si QDs were synthesized via a one-step hydrothermal method. Ultrapure water was used as the blank priming control, while Na_2_SiO_3_ and SiO_2_ NPs were set as reference controls representing different silicon sources. Plate germination assays were first performed to compare the effects of different treatments on maize germination efficiency and early seedling growth potential under NaCl and mixed-salt stress. Subsequently, a pot experiment was conducted using coastal saline soil collected from the Yellow River Delta, to verify the regulatory effects of Si QDs priming on seedling establishment and physiological responses. Specifically, this study aimed to (i) determine whether Si QDs confer greater germination-promoting effects than Na_2_SiO_3_ and SiO_2_ NPs under salt stress; (ii) elucidate the physiological evidence by which Si QDs coordinately enhance early salt tolerance in maize via seed coat interfacial interactions, internalization into seed tissues, ion homeostasis regulation, antioxidant defense activation, and photosynthetic performance modulation. These findings will provide a theoretical basis for the agricultural application of Si QDs in coastal saline farmland in the Yellow River Delta.

## 2 Materials and methods

### 2.1 Synthesis and characterization of Si QDs

Silicon quantum dots (Si QDs) were synthesized using a one-step hydrothermal approach (Na et al., 2019). Detailed procedures are provided in the Supplementary Information (SI, Text S1). Transmission electron microscopy (TEM; JEM-2100F, JEOL, Japan) was used to obtain TEM and high-resolution TEM (HRTEM) images of the Si QDs. Ultraviolet-visible (UV-vis) absorption spectra of the Si QDs aqueous dispersion were recorded over a wavelength range of 200–800 nm using a UV-vis spectrophotometer (UV-9000S, Metash, China). Photoluminescence (PL) spectra were measured using a fluorescence spectrophotometer (F-4600, Hitachi, Japan). For Fourier transform infrared (FTIR) analysis, solid Si QDs samples were mixed with potassium bromide (KBr; SP grade, Sinopharm Chemical Reagent Co., Ltd., Shanghai, China) and pressed into pellets. FTIR spectra were collected over the range of 4000–500 cm^−1^ using an FTIR spectrometer (Tensor 27, Bruker, Germany).

### 2.2 Evaluation of Si QDs on salt tolerance in maize seeds

The mixed-salt solution was prepared to match the typical ionic composition of saline soils in the Yellow River Delta (Fan et al., 2010). The specific ion species and ratios provided in the **Supplementary SI** (**Text S2**). The maize cultivar “Jin Huang Hou 908” (commercially available in Shandong Province, China) was used as the test seed. To identify the optimal priming concentration of Si QDs, we performed concentration gradient screening assays (Xing et al., 2024; Zhuang et al., 2025). Based on the comprehensive evaluation of germination rate (GR), germination potential (GP), germination index (GI), as well as root length, shoot length, and vigor index (VI) of 7-day-old seedlings, we identified 200 mg L^-1^ Si QDs as the optimal priming concentration. Detailed experimental protocols and the corresponding screening results are provided in the **Supplementary SI** (**Text S3**; **Supplementary Figure 1L**, **S2**).

Given that Na_2_SiO_3_, SiO_2_ NPs, and Si QDs exhibit significant differences in their dissolution kinetics, nanoscale size, bioavailability, and effective concentration ranges, we independently determined the optimal priming concentration for each tested material (M. S et al., 2021; Khan et al., 2023; Yang et al., 2024). The applied concentrations for Na_2_SiO_3_ (Feghhenabi et al., 2020; Rahimi et al., 2021) and SiO_2_ NPs (Alsamadany et al., 2024; Zhuang et al., 2025) selected based on previously reported effective priming concentrations for different silicon sources, while the optimal priming concentration for Si QDs was determined based on the preliminary concentration screening assays conducted this study. Four priming treatments were established: hydropriming (H_2_O) as the baseline control, 200 mg L^–1^ Na_2_SiO_3_ as the soluble silicate control, 40 mg L^–1^ SiO_2_ NPs as the conventional nanoparticulate silicon control, and 200 mg L^–1^ Si QDs as the primary experimental intervention. Salinity stress was imposed using individual NaCl and combinatorial mixed-salt regimes, with a concentration gradient set at 0, 25, 50, 75, 100, 125, and 150 mM. According to the soil salinity classification system and electrical conductivity of saturation extract (ECe) ranges proposed by the U.S. Salinity Laboratory (ECe 2–4, 4–8, and 8–16 dS m^-1^ corresponding to slight, moderate, and severe salinization, respectively) (Staff, 1954; Wicke et al., 2011), and in combination with the measured electrical conductivity of the salt solutions at different concentrations used (**Supplementary SI, Table 1**), 25 mM was categorized as a low-salinity level, 50–75 mM as a moderate-salinity level, and 100–150 mM as a high-salinity level, thereby constructing a continuous gradient from low to high salt stress.

During the seed germination assays, the corresponding salt stress conditions for each treatment group were maintained throughout the 7-day incubation period. Seed germination kinetics were monitored every 24 h, and morphological parameters of 7-day-old seedlings were comprehensively quantified at the end of the incubation period (day 7). The experimental procedure was as follows: Maize seeds were primed for 24 hours using the same protocol as in screening experiment (SI, Text S3). After priming, the seeds were placed in Petri dishes (Ø 9 cm) lined with filter paper and containing 3 mL of NaCl solution or mixed-salt solution at the respective concentrations for germination (4 treatments × 2 salt types × 7 salinity levels × 6 replicates × 10 seeds). Dishes were incubated at 25 ± 1 °C in darkness in an artificial climate chamber (RXZ-380C, Ningbo Jiangnan Instrument Factory, China). Germination was recorded daily, defining germination as radicle emergence ≥ 2 mm. GR, GP, and GI were calculated (Sun et al., 2024). On day 7, the shoot and root lengths were measured with a ruler (1 mm minimum division) for all germinated maize seedlings, and the vigor index (VI) was calculated (Yan et al., 2023; Kang et al., 2025).

### 2.3 Surface interactions and uptake of Si QDs by maize seeds

To investigate the interaction of Si QDs with the maize seed coat, the surface morphology and elemental distribution of hydro-primed and Si QDs-primed seeds were characterized using scanning electron microscopy coupled with energy-dispersive X-ray spectroscopy (SEM-EDS) (Prerna et al., 2021; Cappetta et al., 2023). To quantitatively assess the uptake of Si QDs during priming, unprimed, hydro -primed, and Si QDs-primed seeds were dried in an oven at 75 °C for 36 h. Following sequential pre-ashing, high-temperature ashing, and acid digestion, the total silicon content was determined colorimetrically using the molybdenum blue method (Yan et al., 2023; Kang et al., 2025). To further examine the uptake and translocation of Si QDs within maize tissues, the distribution of their intrinsic fluorescence in the roots and shoots of 7-day-old seedlings was visualized using laser scanning confocal microscopy (LSCM, A1Plus, Nikon, Japan) (Haider et al., 2024; Nauman Khan et al., 2024).

### 2.4 Pot experiment

Soil for the pot experiment was collected from the Yellow River Delta Modern Agriculture Comprehensive Experimental Demonstration Base (Dongying, Shandong Province, China; 37°29’ N, 118°61’ E). The soil samples were air-dried, ground, and passed through a 2 mm sieve before use. The soil ECe was 7.32 ± 0.21 dS m^-1^, classifying it as moderately saline, and the other basic physicochemical properties are provided in the SI (**Supplementary Table 2**). The soil moisture was adjusted to 60% of the maximum field water-holding capacity (WHC) with ultrapure water and the soil was then pre-incubated at room temperature for 7 days. To evaluate the effect of Si QDs seed priming on seedling salt tolerance, three treatment groups were established: unprimed seeds sown directly after surface sterilization as the control (CK), hydro-primed seeds, and seeds primed with 200 mg L^−1^ Si QDs. For each treatment, five seeds were sown per pot (8 cm in diameter and 8 cm in height) containing 200 g of the pre-incubated soil, with five replicate pots per treatment. The pot experiment was concluded at 40 days after sowing, at which time all plant growth and salt stress-related physiological parameters were determined. Throughout the experiment, pots were maintained in an artificial climate chamber (RXZ-380C, Ningbo Jiangnan Instrument Factory, China) under controlled conditions of 25 ± 1 °C with a 12 h light/12 h dark photoperiod. Soil moisture was maintained at 60% of WHC by daily gravimetric watering, and pots were randomly repositioned at regular intervals to minimize potential spatial heterogeneity within the chamber.

### 2.5 Measurement of seedling physiological parameters

At the end of the pot experiment, photosynthetic parameters were measured immediately. Between 9:00 and 11:00 a.m., the net photosynthetic rate (P_n_), transpiration rate (T_r_), instantaneous water use efficiency (IWUE, calculated as P_n_/T_r_), stomatal conductance (G_s_), and intercellular CO_2_ concentration (C_i_) of fully expanded leaves were measured using a portable photosynthesis system (CIRAS-3, PP Systems, USA) (Haider et al., 2024). The instrument was calibrated according to the manufacturer’s instructions before measurement. Leaf chlorophyll relative content (SPAD) was determined using a chlorophyll meter (TYS-4N, Jinkelida, China). The whole plants were then carefully excavated, and the roots were washed with ultrapure water and blotted dry with filter paper. Plant height and stem diameter were measured using a ruler and a vernier caliper, respectively. The plants were separated into shoots and roots, and the fresh weights of shoots and roots were recorded using an electronic balance. Root images were obtained using a root scanner (10000XL, Epson, Japan), and root morphological parameters, including root length (RL), root average diameter (RD), root area (RA), and root volume (RV), were analyzed using WinRHIZO software (Pro. 2005, Regent Instruments, Canada) (Yin et al., 2025). Root vigor (RVI) was determined using the triphenyltetrazolium chloride (TTC) reduction method (Li et al., 2023b).

A portion of the fresh samples was used to determine leaf superoxide dismutase (SOD) activity and malondialdehyde (MDA) content using commercial assay kits (Cat. No. A001-3–2 and A003-1-2, respectively; Nanjing Jiancheng Bioengineering Institute, China) following the manufacturer’s instructions (Dong et al., 2025). Another portion of the fresh samples was heated at 105 °C for 1 h and then oven-dried at 45 °C to constant weight. The dried roots and leaves were digested separately with H_2_SO_4_ and H_2_O_2,_ and the concentrations of Na^+^ and K^+^ in roots and leaves were determined using an atomic absorption spectrophotometer (AAS; TAS-990 AFG, Purkinje General, China) (Liu et al., 2021).

### 2.6 Statistical analysis

Statistical analysis was performed on Statistical Product and Service Solutions Software (Version 26.0, SPSS Inc, Chicago, IL, USA). All the data were analyzed by Duncan’s multiple range test, and statistical significance was defined as *P* < 0.05. The results were expressed as the means ± standard deviation (SD). Mantel test was used to analyze the relationships of seedling photosynthetic performance, antioxidant defenses, and the Na^+^/K^+^ ratio with maize seedling biomass and plant morphology. Based on the Mantel test results, variables showing significant correlations with biomass and morphology were selected to construct a structural equation model (SEM) to evaluate the direct and indirect effects of Si QDs on maize physiological parameters. Model adequacy was assessed using a non-significant chi-square (χ^2^) test and high goodness-of-fit indices. Further details are provided in the SI (Text S4).

## 3 Results

### 3.1 Characteristics of Si QDs

**Figure 1:**
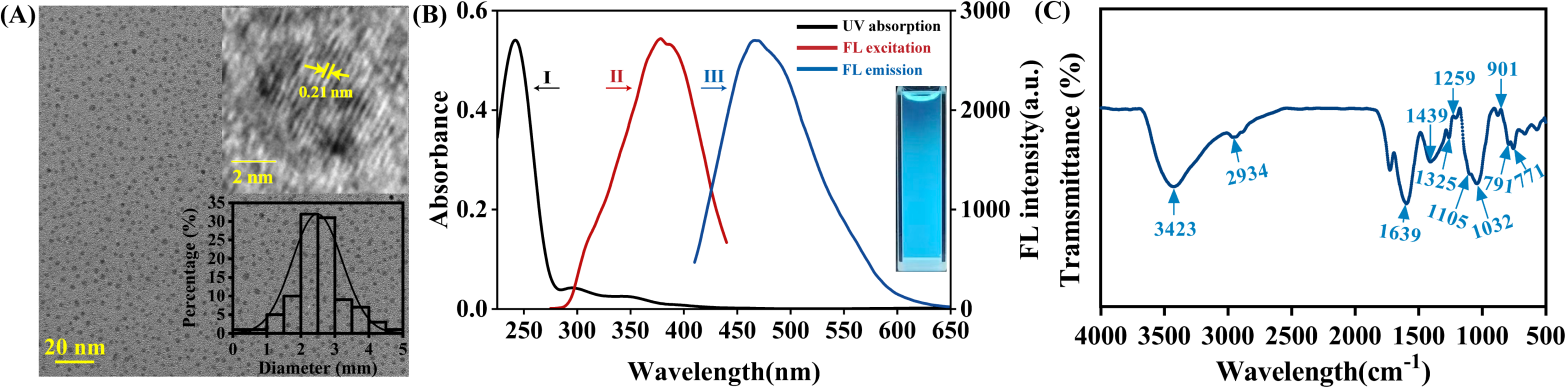
Characterization of Si QDs. **(A)** TEM image of Si QDs, scale bar = 20 nm (Inset: HRTEM image and size distribution of Si QDs). **(B)** UV–Vis absorption (I), fluorescence excitation (II), and emission spectra (III) (Inset: Si QDs solution under 365 nm UV light). **(C)** FTIR spectrum of Si QDs.

**Figure 1** shows the TEM image of the synthesized Si QDs. The Si QDs were quasi-spherical and well-dispersed, with a relatively uniform size distribution and an average particle diameter of 2.48 ± 0.74 nm. The HRTEM image showed a lattice fringe with a spacing of 0.21 nm, confirming the crystalline nature of the Si QDs. **Figure 1** illustrates the UV-Vis absorption spectrum (Curve I) and the fluorescence spectra (curves II and III) of the Si QDs. As shown by curve I, absorption peaks were observed at 298 nm and 350 nm, corresponding to the π–π* transition of C=C and the n–π* transition of C=O, respectively. The fluorescence spectra showed that, under excitation at 390 nm, the Si QDs exhibited a maximum emission peak at 460 nm (curve III). In addition, the excitation spectrum monitored at an emission wavelength of 460 nm showed a profile similar to that of the emission spectrum (curve II). Meanwhile, the Si QDs suspension emitted blue fluorescence under 365 nm UV irradiation (**Figure 1**, inset), confirming the photoluminescent properties of the synthesized Si QDs.

FTIR analysis further revealed the surface functional groups of the Si QDs (**Figure 1**). The absorption band at 1105 cm^-1^ was associated with the stretching vibration of Si–O–Si. The peak at 1032 cm^-1^ was attributed to the asymmetric deformation vibration of Si–C, and the signal at 901 cm^-1^ indicated the presence of Si–H stretching vibrations. The absorption peak at 2934 cm^-1^ corresponds to the asymmetric stretching vibration of C–H, the strong signal at 1639 cm^-1^ was assigned to C=O stretching vibrations, and the broad peak at 1439 cm^-1^ originated from C–O vibrations. The broad absorption band at 3423 cm^-1^ was assigned to N–H stretching vibrations and may represent overlapping unresolved peaks of primary and secondary amines. The presence of amine groups was further supported by the C–NH bending vibration at 1259 cm^-1^ and N–H rocking vibrations at 771–796 cm^-1^. Additionally, the characteristic peak at 1325 cm^-1^ corresponded to O–H bending vibrations. Collectively, these FTIR results demonstrate that the surface of the Si QDs is enriched with hydrophilic functional groups, particularly amino and hydroxyl moieties, which underpin their excellent water dispersibility. In summary, the above characterizations confirm the successful synthesis of Si QDs possessing a nanoscale size, intrinsic photoluminescence, and favorable water dispersibility.

### 3.2 Si QDs seed priming improved the seed germination performance under salt stress

**Figure 2:**
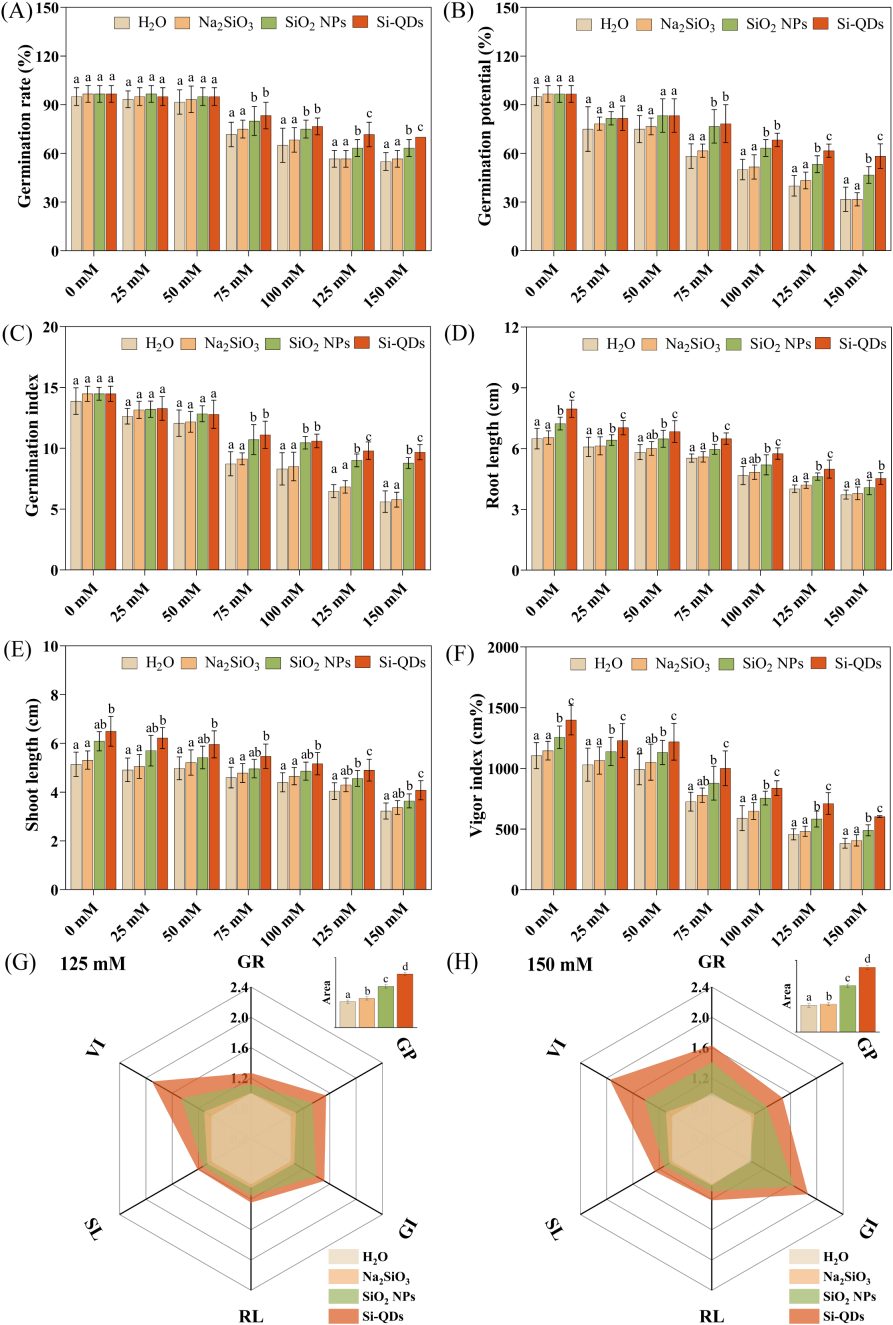
Effect of seed priming with Si QDs on maize seed germination under mixed-salt stress. **(A)** Germination rate, **(B)** Germination potential, **(C)** Germination index, **(D)** Shoot length, **(E)** Root length, **(F)** Vigor index, The comprehensive effects of Si QDs on seed germination under 125mM **(G)** and 150 mM **(H)** mixed-salt stress. Data are means ± the standard deviation (*n* = 6). The different small letters reflect a significant difference among the different treatments (Duncan’s multiple-comparison test, *P* < 0.05).

**Figure 3:**
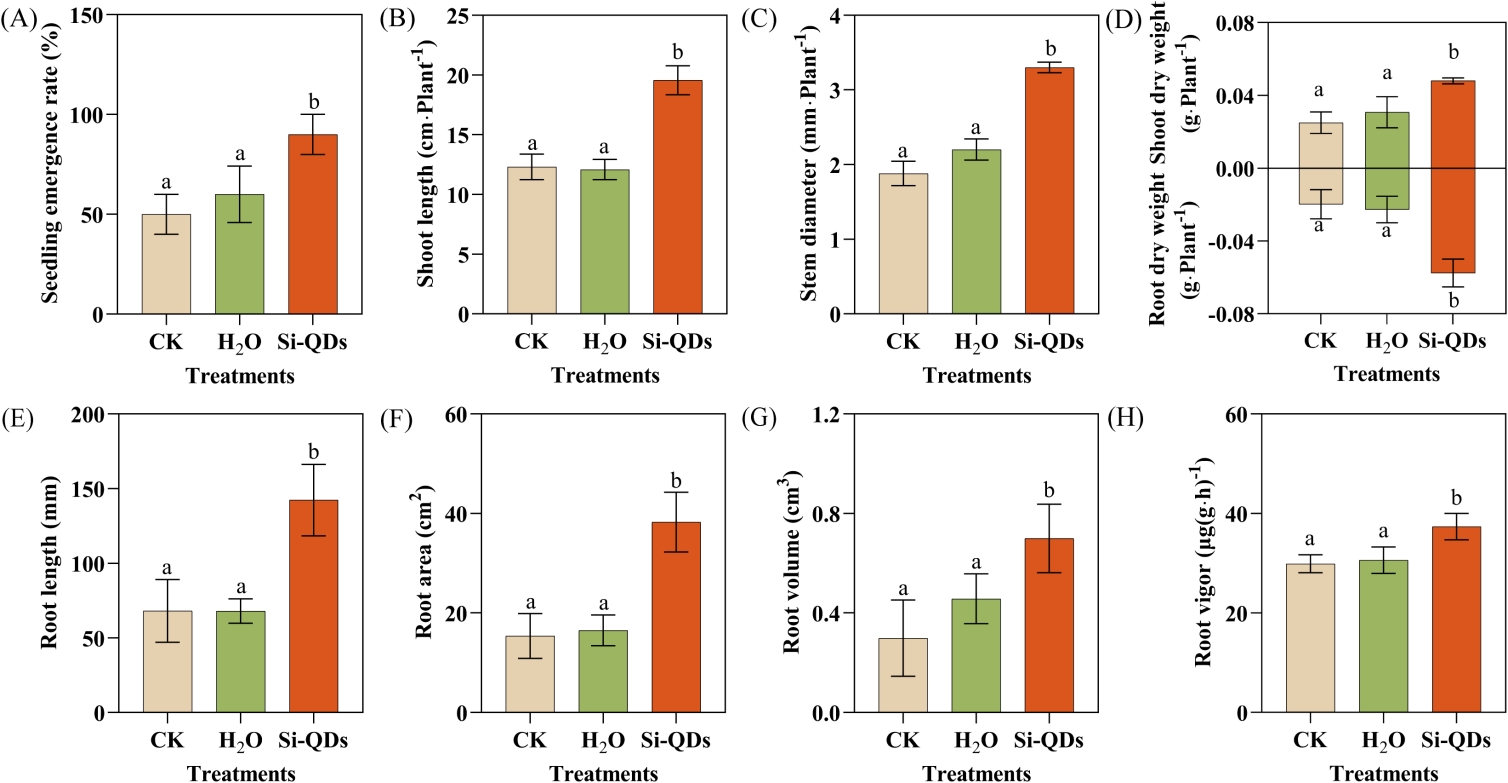
Effect of seed priming with Si QDs on maize seedling growth. **(A)** Seedling emergence rate, **(B)** Shoot length, **(C)** Stem diameter, **(D)** Shoot and root dry weight, **(E)** Root length, **(F)** Root area, **(G)** Root volume, **(H)** Root vigor. Data are means ± the standard deviation (*n* = 5). The different small letters reflect a significant difference among the different treatments (Duncan’s multiple-comparison test, *P* < 0.05).

To systematically evaluate the effects of Si QDs priming on maize germination under salt stress and to distinguish its efficacy from that of other silicon sources, germination assays were conducted under both mixed-salt (**Figure 2**) and NaCl (**Figure 3**) stress. Hydropriming (H_2_O) served as the blank control, while sodium silicate (Na_2_SiO_3_) and silica nanoparticles (SiO_2_ NPs) were employed as comparative controls representing distinct silicon sources. At low salinity (≤ 50 mM), no significant differences were observed among treatments in GR, GP, or GI (**Figure 2A**). Moreover, GR remained close to 100% across all treatments, indicating that the 200 mg L^-1^ Si QDs priming solution exhibited negligible nanotoxicity toward maize seeds under the tested conditions. Conversely, as the mixed-salt concentration increased to ≥ 75 mM, seed germination was progressively inhibited across all treatments, with the severity of suppression exacerbating in a dose-dependent manner (**Figure 2A**). Importantly, the Si QDs-primed seeds exhibited significantly higher GR, GP, and GI compared to the hydro-primed (*P* < 0.05), with increases of 16.3–27.3%, 34.3–84.2%, and 27.4–72.6%, respectively. Further comparison among different silicon source treatments showed that Si QDs had a stronger germination-promoting effect under high salt stress. Compared with Na_2_SiO_3_ priming, Si QDs priming significantly increased GR, GP, and GI by 11.1–23.5%, 27.1–84.2%, and 21.7–67.6%, respectively, at mixed-salt concentrations of ≥ 75 mM (*P* < 0.05, **Figure 2A**). Compared with SiO_2_ NPs priming, Si QDs priming significantly increased GR, GP, and GI by 10.5–13.2%, 15.6–25.0%, and 8.8–10.2%, respectively, at mixed-salt concentrations of ≥ 125 mM (*P* < 0.05, **Figure 2A**). Collectively, these findings indicate that under high salt stress, Si QDs priming is more effective than either hydropriming or priming with other silicon sources in improving maize seed germination.

Further evaluation of seedling morphological traits revealed that across the mixed salt concentration range of 0–150 mM, Si QDs priming consistently and significantly enhanced root length (RL), shoot length (SL), and the vigor index (*P* < 0.05, **Figure 2D**). Compared with hydropriming, Si QDs priming resulted in notable increases in RL (10.1–24.3%), SL (18.9–26.4%), and VI (46.0–95.0%) (*P* < 0.05). Relative to Na_2_SiO_3_ priming, Si QDs priming yielded significant enhancements of 9.2–21.7% for RL, 11.4–21.5% for SL, and 32.5–77.9% for VI (*P* < 0.05). When compared with SiO_2_ NPs priming, Si QDs priming significantly augmented RL by 4.40–10.9% and VI by 3.2–37.3% (*P* < 0.05). Radar charts were generated using the normalized values of GR, GP, GI, RL, SL, and VI to comprehensively assess the overall germination-promoting efficacy of each treatment under high salt stress conditions (125 and 150 mM) (**Figure 2G**). Multivariate integration via radar chart analysis revealed the overall high-efficiency of Si QDs priming, which significantly outperformed Na_2_SiO_3_ and SiO_2_ NPs by 132.3% and 39.9%, respectively (*P* < 0.05). Collectively, these findings demonstrate that Si QDs priming robustly promotes seed germination efficiency and early seedling establishment under high salt stress.

Under NaCl stress, Si QDs priming exhibited an overall pattern consistent with that observed under mixed-salt stress (**Figure 3**). At NaCl concentrations ≤ 25 mM, there were no significant differences in GR, GP, or GI among treatments (**Supplementary Figure 3A**). When NaCl concentration was ≥ 50 mM, Si QDs priming significantly increased GR, GP, and GI compared with hydropriming and Na_2_SiO_3_ priming (*P* < 0.05). Relative to SiO_2_ NPs priming, Si QDs priming significantly improved GR, GP, and GI at NaCl concentrations ≥ 100 mM (*P* < 0.05). Across the full NaCl concentration range of 0–150 mM, Si QDs priming also significantly increased root and shoot lengths and vigor index compared with other treatments (*P* < 0.05; **Supplementary Figure 3D**).

### 3.3 Si QDs seed priming promoted maize seedling growth in coastal saline soils

**Figure 4:**
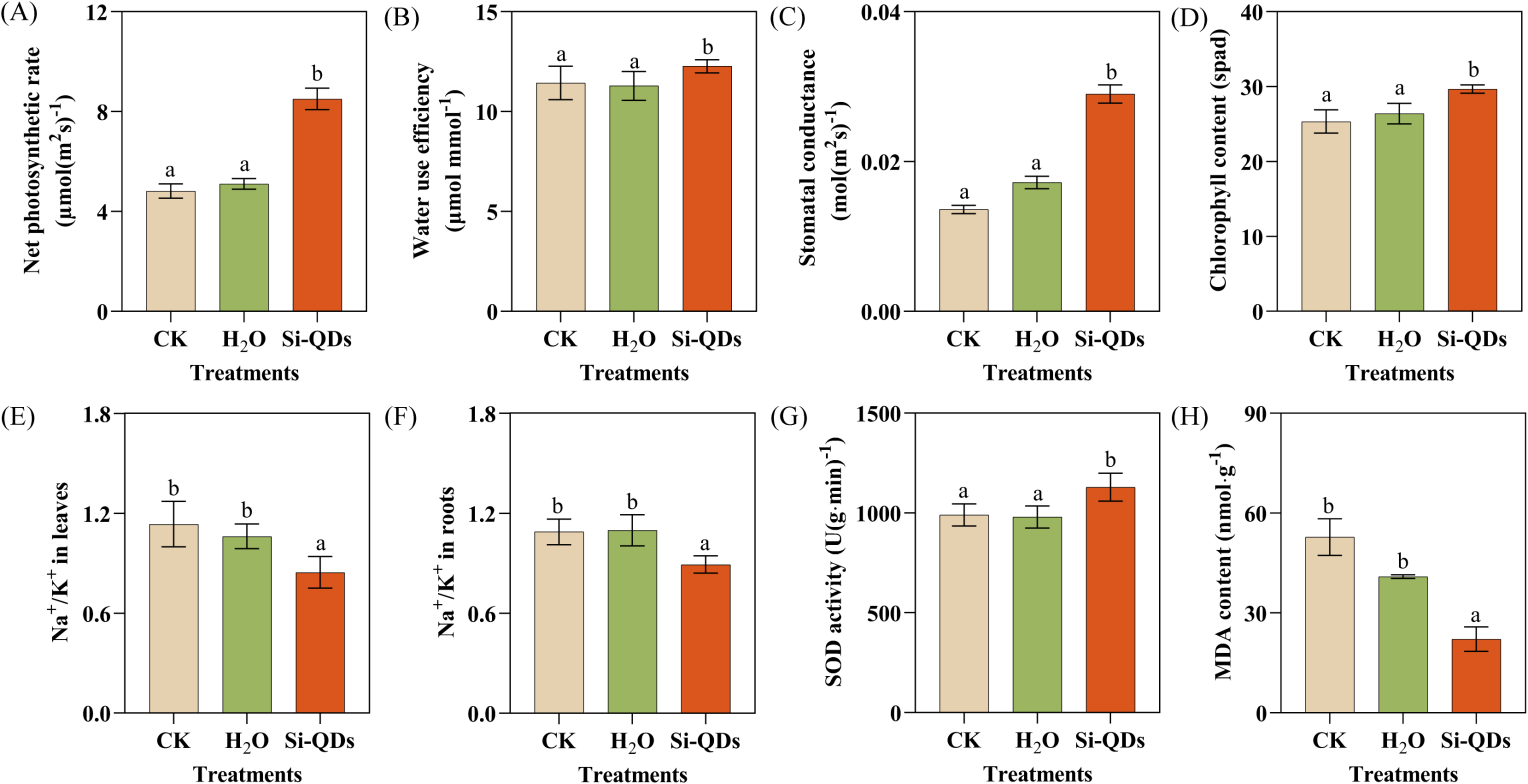
Effect of seed priming with Si QDs on physiological characteristics in maize seedlings. **(A)** Net photosynthetic rate, **(B)** Transpiration rate, **(C)** Stomatal conductance, **(D)** Chlorophyll content, **(E)** Na^+^/K^+^ in leaves, **(F)** Na^+^/K^+^ in roots, **(G)** SOD activity, **(H)** MDA content. Data are means ± the standard deviation (*n* = 5). The different small letters reflect a significant difference among the different treatments (Duncan’s multiple-comparison test, *P* < 0.05).

**Figure 5:**
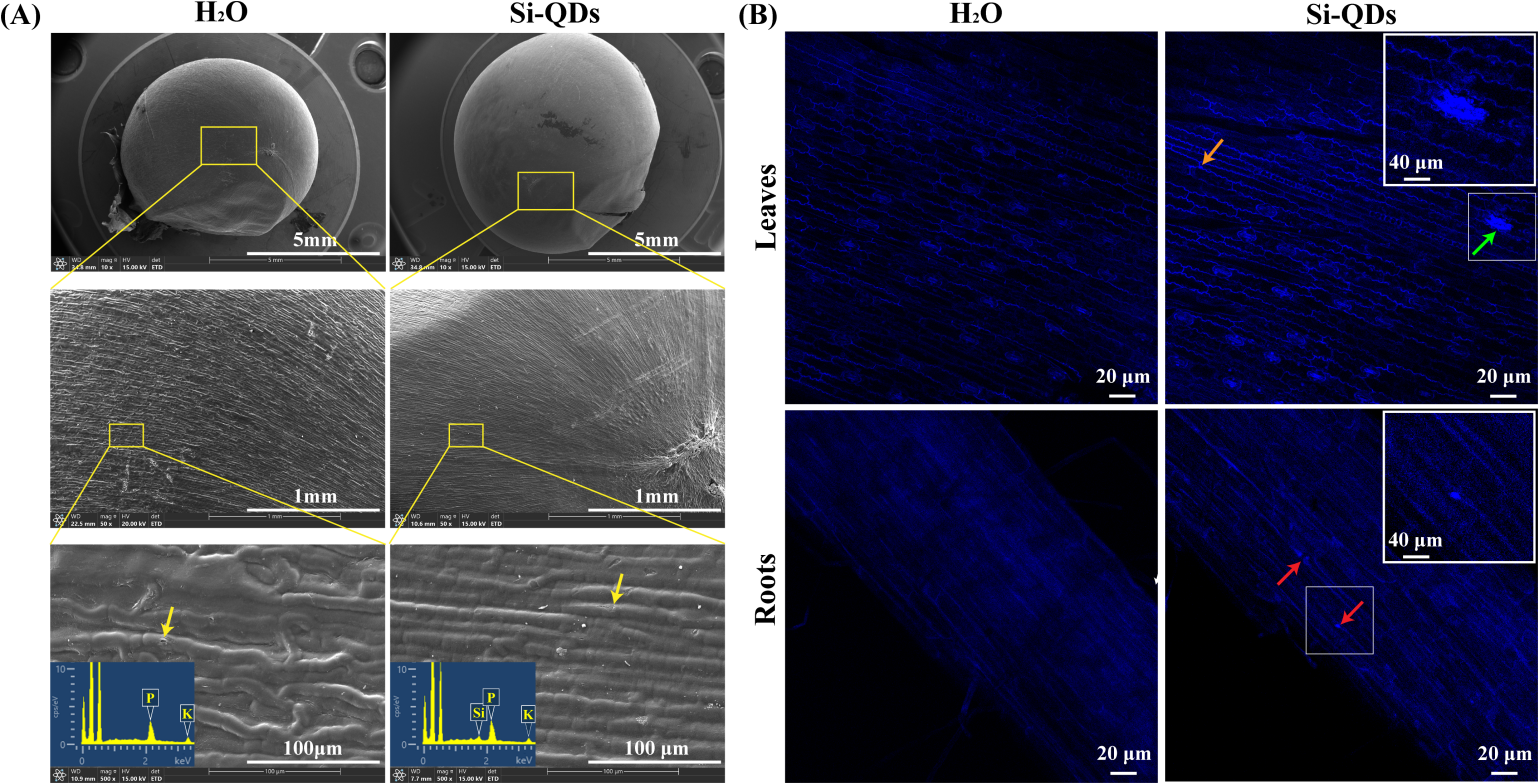
Internalization and transport of Si QDs in maize seeds and seedlings. **(A)** SEM images of outer (top) surfaces of maize seed tegument with seeds primed with water or Si QDs, **(B)** Laser confocal microscope images of cotyledons and roots from 7-day-old maize seedlings with seeds primed with water or Si QDs.

The preliminary germination experiments showed that the growth-promoting efficacy of Si QDs seed priming was most evident under moderate and high salt stress levels. Therefore, a pot experiment was performed using the moderately saline soil (7.32 ± 0.21 dS m^-1^) which has agricultural development potential and was collected from the Yellow River Delta, to evaluate the effects of Si QDs seed priming on maize seedling growth (**Figure 3**; **Figure 4**). Hydropriming had no significant effects on seedling growth compared with the CK (*P* > 0.05). Conversely, Si QDs seed priming significantly enhanced the seedling emergence rate, shoot length, and stem diameter, outperforming both the CK and hydropriming. Specifically, these morphological parameters were 1.5-, 1.6-, and 1.5-fold higher than those of the hydro-primed seedlings, respectively (*P* < 0.05; **Figure 3A**). Furthermore, relative to hydropriming, the shoot and root fresh weights of the Si QDs-primed maize increased by 50.1% and 143.9% (*P* < 0.05; **Supplementary Figure 4**), while the corresponding dry weights were elevated by 56.1% and 153.7%, respectively (*P* < 0.05; **Figure 3**). Hydropriming did not significantly alter root morphology relative to the CK (*P* > 0.05). In contrast, compared with hydropriming, Si QDs seed priming significantly increased root length, root area, and root volume by 109.4%, 131.8%, and 53.1%, respectively (*P* < 0.05; **Figure 3E**), while the average root diameter remained unchanged (**Supplementary Figure 4**). Concurrently, Si QDs seed priming significantly enhanced root vigor (*P* < 0.05; **Figure 3**). Comprehensive evaluation via radar chart analysis (**Figure 5**) showed that, compared with CK and hydropriming, the composite growth index of seedlings derived from Si QDs-primed seeds was remarkably enhanced by 237.51% and 227.68%, respectively.

### 3.4 Si QDs seed priming regulated the physiological characteristics of maize in coastal saline soils

To systematically investigate the effects of Si QDs seed priming on the physiological traits of maize seedlings cultivated in coastal salinized soils, a comparative analysis was conducted encompassing photosynthetic performance, Na^+^ and K^+^ homeostasis, SOD activity, and MDA content (**Figure 4**; **Supplementary Figure 6**, **S7**). Consistent with previous morphological observations, hydropriming exerted no significant influence on the photosynthetic parameters relative to the CK (*P* > 0.05). In contrast, Si QDs seed priming markedly optimized the overall photosynthetic capacity under salt stress. Specifically, the net photosynthetic rate (P_n_; **Figure 4**), transpiration rate (T_r_; **Figure 4**), stomatal conductance (G_s_; **Figure 4**), and instantaneous water use efficiency (IWUE; **Supplementary Figure 6**) of the Si QDs-primed seedlings were substantially elevated compared to both the CK and hydropriming (*P* < 0.05). Relative to hydropriming, Si QDs seed priming yielded enhancements of 66.69% in P_n_, 52.8% in T_r_, 69.1% in G_s_, and 8.7% in IWUE. Furthermore, Si QDs seed priming significantly increased the relative leaf chlorophyll content (SPAD value) by 12.4% (*P* < 0.05; **Figure 4**), while driving a significant 9.9% reduction in the intercellular CO_2_ concentration (C_i_) (*P* < 0.05; **Supplementary Figure 6**). Si QDs seed priming profoundly regulated ion homeostasis across both root and foliar tissues. Compared to hydropriming, Si QDs seed priming significantly restricted Na^+^ accumulation by 9.9% in the leaves and 8.5% in the roots (*P* < 0.05; **Supplementary Figure 7A**). Simultaneously, it facilitated essential K^+^ assimilation, increasing K^+^ concentrations by 13.4% and 11.9% in the respective tissues (*P* < 0.05; **Supplementary Figure 7B**). Consequently, the critical Na^+^/K^+^ ratios in the leaves and roots were substantially attenuated by 20.4% and 18.7%, respectively (*P* < 0.05; **Figure 4E**). The effects of Si QDs seed priming on SOD activity and MDA content in maize seedling leaves are shown in (**Figures 4G, H**). Compared with hydropriming, Si QDs seed priming significantly increased SOD activity (10.1%) and decreased MDA content (25.4%) (*P* < 0.05; **Figure 4G**).

## 4 Discussion

The growth-promoting efficacy of Si QDs seed priming on maize germination and early seedling establishment was primarily observed under moderate to high salt stress, with a significantly greater enhancement magnitude than hydropriming, Na_2_SiO_3_, and SiO_2_ NPs priming (**Figure 2**). This indicates that the efficacy of Si QDs under high salinity arises from the nanomaterials’ unique physicochemical properties, which only take effect under the high osmotic and ionic stress of saline conditions (Khan et al., 2023). Under salt stress, seed imbibition, reserve mobilization, and radicle protrusion are suppressed by osmotic stress and ion-specific toxicity (Chen et al., 2023). Nanomaterials can enhance germination efficiency by promoting seed imbibition, regulating phytohormone and redox homeostasis, and inducing transcriptional reprogramming in seeds (Ulhassan et al., 2023; Salam et al., 2025; Ulhassan et al., 2025). Importantly, material size, surface chemistry, and stability under saline conditions are key factors governing these processes (Khan et al., 2023; Soni et al., 2026). Compared with Na_2_SiO_3_ and SiO_2_ NPs, Si QDs possess an ultrasmall particle size, superior aqueous dispersibility, and abundant hydrophilic surface functional groups (**Figure 1**). Collectively, these physicochemical attributes likely constitute the material basis for the observed advantage of Si QDs over other silicon sources under saline conditions.

First, the internalization of nanomaterials into seed tissues is inherently limited by the pore size of the crop cell wall (5–20 nm), and smaller particles are more capable of traversing these pores and translocating within tissues (Seleiman et al., 2021). The Si QDs synthesized in this study had an average particle size of only 2–3 nm (**Figure 1**), which is markedly smaller than that of conventional SiO_2_ NPs. This ultrasmall size theoretically enables efficient translocation across the seed coat and cell wall barriers, allowing the nanomaterials to directly reach the embryonic axis and tissues of the emerging seedling. Second, the high ionic strength in saline environments typically reduces the electrostatic repulsion between nanoparticles and induces particle agglomeration, which in turn impairs their mobility and bioavailability in plant tissues (Lee et al., 2025). The Si QDs synthesized in the study are rich in hydrophilic functional groups (e.g., hydroxyl and amino groups, **Figure 1**). These groups confer better aqueous dispersibility on the nanomaterials, and enhance their ability to form stable interfacial contact with the seed coat surface. Indeed, SEM-EDS analysis demonstrated that Si-QD priming induced topographical changes on the seed coat and resulted in localized silicon deposition (**Figure 5**), confirming significant interfacial interactions between Si QDs and seed coat. Crucially, these dynamic interactions may, on one hand, enhance localized wettability to accelerate seed imbibition, and on the other, form a physical exclusion barrier that effectively restricts the apoplastic influx of Na^+^ into seeds during the initial stages of germination (E et al., 2024). Furthermore, LSCM imaging combined with total silicon quantification demonstrated that Si QDs were successfully internalized into, and translocated within, the root and shoot tissues of 7-day-old maize seedlings (**Figure 5**, **Supplementary S8**). These results collectively verify that Si QDs enhance the early salt tolerance of maize seeds in saline environments via interfacial contact with the seed coat and efficient tissue penetration. In contrast, the other tested silicon sources exert their biological effects likely via different mechanisms. As a soluble silicate source, Na_2_SiO_3_ depends heavily on specific silicon transport systems (e.g., Lsi transporters) and on silicon speciation and biotransformation in the rhizosphere and within tissues, the bioavailability of which is severely compromised under adverse environmental stresses such as salinity (Zhu et al., 2019). Conversely, SiO_2_ NPs may exhibit attenuated priming efficacy due to their larger particle dimensions and a pronounced propensity to undergo agglomeration under high ionic strength conditions (Lee et al., 2025).

The growth-promoting effects of Si QDs seed priming extended beyond the germination stage, and persisted throughout the seedling establishment phase in coastal saline soil (**Figure 3**). In this study, Si QDs seed priming significantly increased the emergence rate, plant height, stem diameter, and biomass of maize, confirming that the beneficial effects conferred during the germination stage can be effectively translated into vigorous early seedling growth. Previous studies have shown that seed priming can enhance seedling establishment by pre-activating germination-related metabolic pathways, improving cellular repair capacity, and increasing environmental adaptability, which enables seeds to initiate growth more rapidly after sowing (Alsamadany et al., 2024; Zhuang et al., 2025). Concurrently, Si QDs seed priming significantly increased root length, root surface area, root volume, and root vigor in maize seedlings, indicating that this treatment promoted the formation of a robust early root system architecture. This optimized root system likely enhanced the foraging capacity of maize seedlings, maximizing the uptake efficiency of water, essential mineral ions, and limited soil nutrients, thereby maintaining normal plant development and growth under salt stress (Sun et al., 2021; Dong et al., 2025).

The optimization of ion homeostasis may represent a key mechanism by which Si QDs promote seedling growth. Under salt stress, excessive cytosolic Na^+^ accumulation disrupts intracellular ion balance and impairs critical physiological and metabolic processes (Pompelli et al., 2021; Zahra et al., 2022), whereas a high capacity to retain K^+^ is essential for maintaining cytosolic pH homeostasis, supporting protein synthesis, and enabling accurate stomatal regulation (Lebaudy et al., 2007; Wang and Wu, 2010). Si QDs seed priming significantly reduced Na^+^ concentrations and increased K^+^ concentrations in both leaf and root tissues of maize seedlings (**Figure 4**), thereby effectively alleviating salt-induced ionic imbalance and preserving normal seedling growth (Ali et al., 2021). Previous studies have shown that the regulation of ionic homeostasis by exogenous silicon is often closely associated with the activation of salt−responsive transport systems. Silicon can upregulate the expression of genes such as *SOS1*, which is involved in plasma membrane Na^+^ extrusion, and *HKT1*, which mediates selective Na^+^/K^+^ transport, thereby coordinately enhancing Na^+^ exclusion or compartmentalization and improving K^+^ retention efficiency (Gharbi et al., 2025). In addition, silicon can increase the transcript abundance of aquaporin genes such as *NtPIP1;1*, thus enhancing root hydraulic conductivity and improving water uptake and membrane homeostasis (Liu et al., 2024b). Given that Si QDs successfully internalize into maize root and foliar tissues, their capacity to confer salt tolerance likely involves the synergistic regulation of ion and water transport networks. This precise molecular regulation optimizes ion uptake selectivity and root hydraulic efficiency, ultimately maintaining Na^+^/K^+^ homeostasis (Sarkar et al., 2022).

In addition to optimizing ion homeostasis, alleviating oxidative stress represents another key mechanism by which Si QDs promotes early seedling establishment. Salt stress is commonly accompanied by the overproduction of ROS and exacerbated lipid peroxidation, which collectively compromise cellular membrane integrity and impair essential metabolic processes (Sun et al., 2020). Si QDs seed priming significantly upregulated SOD activity and reduced MDA accumulation in maize seedling leaves (**Figure 4**), indicating a marked enhancement of the antioxidant defense system and a pronounced decrease in oxidative damage under salt stress. This enhancement of antioxidant defense aligns with observations in maize seedlings treated with foliar Si QDs (Fan et al., 2023). Previous studies have demonstrated that soil application of Si QDs upregulates the expression of antioxidant enzyme-related genes, including *SOD*, *CAT*, and *POD*, in maize (Xiao et al., 2023). Aligning with the observed enhancements in SOD activity and the reduction in MDA content, we postulate that the salt tolerance conferred by Si QDs relies on the transcriptional upregulation of the antioxidant defense machinery. However, this hypothesis remains to be further verified. From a materials chemistry perspective, the abundant hydroxyl (-OH) moieties present on the surface of the Si QDs synthesized herein may endow the nanomaterials with intrinsic ROS-scavenging capabilities (**Figure 1**). Previous studies on carbon-based nanomaterials have established that surface -OH groups can directly scavenge free radicals by transferring unpaired electrons to the delocalized C–C backbone (Li et al., 2020a). Collectively, the pronounced antioxidant effects of Si QDs seed priming may be attributed to a synergistic dual mechanism that involves the elicitation of the plant’s endogenous antioxidant system coupled with the direct, nanomaterial-mediated chemical scavenging of ROS.

**Figure 6:**
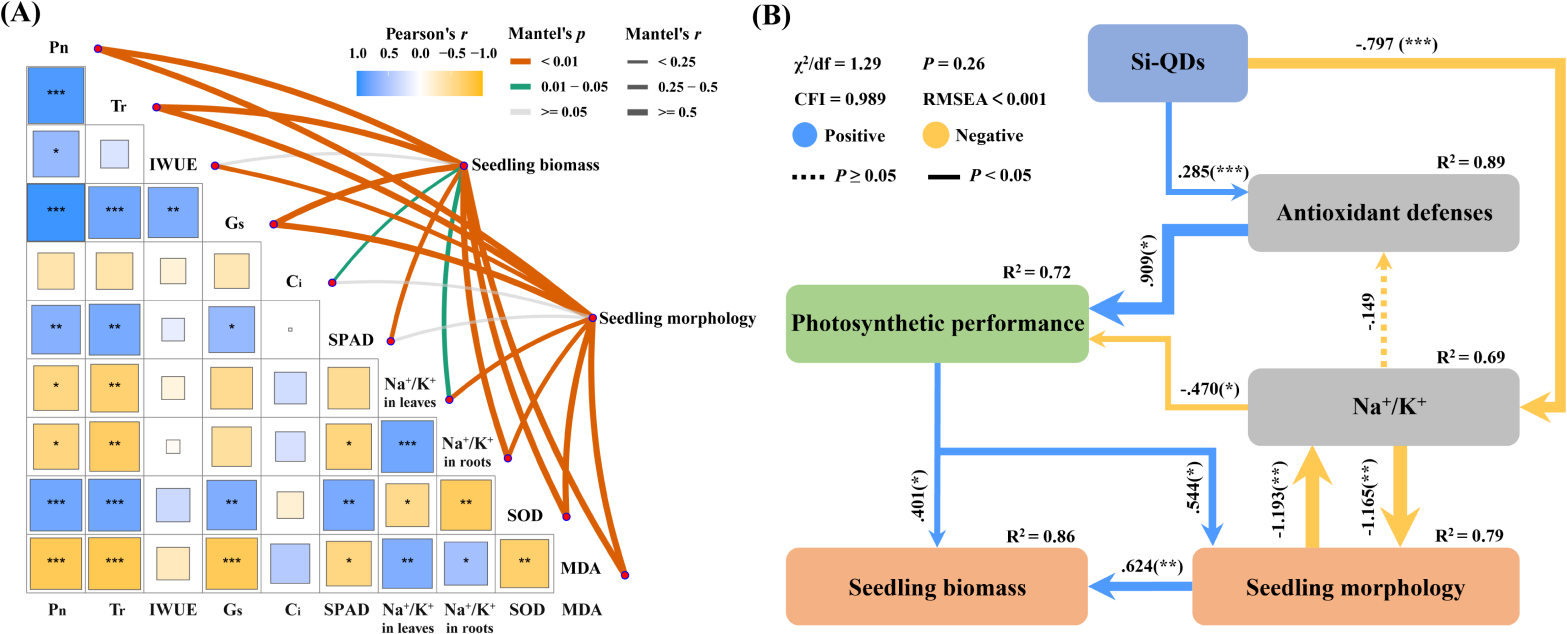
Regulatory factors and response rules of seed priming with Si QDs to alleviate salt stress in maize seedlings. **(A)** Mantel test, the color gradient representing the Pearson correlation coefficient, Line width corresponding to Mantel’s r value and line color indicating statistical significance. **(B)** Structural equation modeling (SEM) showing the effects of Si QDs on antioxidant defenses, Na^+^/K^+^, photosynthetic performance, seedling biomass, and seedling morphology, solid line indicates a significant effect and dotted line indicates a nonsignificant effect, numbers above the arrow lines are path coefficient, *R*^2^ indicates the variance of the dependent variable explained by the model, the width of the arrows indicates the value of the path coefficient, significance level: * denoting *P* < 0.05, ** indicating *P* < 0.01, *** marking *P* < 0.001, blue and yellow lines indicate positive and negative coefficients, respectively.

By optimizing ion homeostasis and strengthening antioxidant defense in seedlings, Si QDs seed priming significantly enhanced photosynthetic performance, which may serve as a fundamental physiological basis for its promotion of salt tolerance. In the present study, Si QDs seed priming significantly increased the Pn, Tr, IWUE, Gs, and SPAD in maize seedling leaves, while decreasing Ci. This substantial enhancement of photosynthetic performance is consistent with findings in maize treated with Si NPs (Rizwan et al., 2023). An attenuated Na^+^/K^+^ ratio helps maintain cellular ion homeostasis and stomatal regulation, whereas the alleviation of oxidative damage preserves chloroplast structure and the stability of the photosynthetic apparatus (Chai et al., 2024; Zhang et al., 2025). Together, these effects culminate in a higher photosynthetic capacity. Additionally, previous studies have suggested that Si QDs can function as artificial antenna, wherein their blue emission under UV excitation can amplify foliar light-harvesting capacity and thereby improve plant photosynthesis (Li et al., 2020b). Integrated Mantel test and structural equation modeling analyses further substantiated these relationships (**Figure 6**). The Na^+^/K^+^ ratio, SOD activity, and MDA content were each significantly correlated with photosynthetic parameters (*P* < 0.05), which in turn were significantly correlated with seedling biomass and morphology. Collectively, these findings reveal a close physiological coupling among optimized ion homeostasis, alleviated oxidative damage, and enhanced photosynthesis. They corroborate the mechanistic paradigm that Si QDs seed priming enhances salt tolerance in maize seedlings by maintaining Na^+^/K^+^ homeostasis and elevating antioxidant capacity, thus mitigating salt-induced growth inhibition and ultimately promoting biomass accumulation and root system development.

Notably, although results from petri dish and pot experiments confirm that Si QDs seed priming enhances ion homeostasis, antioxidant defense, and photosynthetic performance in maize, its agronomic effectiveness under realistic saline field conditions remains to be validated. In saline farmlands, crop seedling establishment is highly sensitive to environmental fluctuations, particularly salt redistribution driven by rainfall and irrigation, as well as dynamic changes in soil moisture and temperature (Islam, 2025). Nanomaterials may also undergo aggregation, aging, and environmental transformation, which can alter their bioavailability and the durability of their effects (Jamei et al., 2026). Previous field studies have shown that the effects of silicon-based nanomaterials on crop performance vary considerably with irrigation regimes, soil nutrient status, and crop genotype, indicating that their field efficacy is modulated by environment (Ding et al., 2022; Liang et al., 2026). Whether the benefits of Si QDs seed priming can ultimately be translated into tangible agronomic gains (e.g., yield, quality, or nutritional improvement), whether supplementary agronomic practices (e.g., foliar application) are required at later growth stages, and how its ecological safety should be systematically evaluated, all warrant further in-depth investigation (Hofmann et al., 2020; Khan et al., 2025). Therefore, the actual field efficacy, applicable scope, and safety thresholds of Si QDs seed priming must be systematically evaluated via multi-location, multi-season, full crop life-cycle field trials.

## 5 Conclusions

As a seed priming agent for maize (*Zea mays* L.), Si QDs exerted stronger germination-promoting effects under high salt stress compared to Na_2_SiO_3_ and SiO_2_ NPs. Crucially, these priming benefits were sustained from the germination stage to seedling establishment. Physiological analyses further indicated that, due to their ultrasmall particle size and abundant hydrophilic functional groups, Si QDs could adhere more effectively to the seed coat and be internalized into seed tissues more efficiently, thereby enhancing maize germination. In addition, Si QDs enhanced the salt tolerance of maize seedlings by improving ion homeostasis, alleviating oxidative damage, and maintaining photosynthetic capacity. Compared with the more commonly reported application modes of soil application and foliar spraying, this study extended the application of Si QDs to seed priming, confirmed their relative advantages as priming materials, and provided a theoretical basis for their potential application in coastal saline soil of the Yellow River Delta. Future studies should combine qPCR, transcriptomic analysis, and proteomic analysis to further elucidate the specific molecular regulatory mechanisms by which Si QDs enhance maize salt tolerance. Meanwhile, field experiments under actual environments should be conducted, including multi-location, multi-season, and full crop life-cycle trials, to systematically assess the stability, applicability, and ecological safety of Si QDs in saline farmland ecosystems.

## Data Availability

The raw data supporting the conclusions of this article will be made available by the authors, without undue reservation.
