# Supplementary material for: Seed priming with silicon quantum dots promotes maize seedling establishment in coastal saline soil
Source: Front Plant Sci. 2026 Apr 14;17:1796045. doi: 10.3389/fpls.2026.1796045 (PMC13120962; doi:10.3389/fpls.2026.1796045)
Supplement: Supplementary file 1 [file SupplementaryFile1.docx]

***Supplementary Information***

**Table of Contents**

[Text S1. Preparation of Si-QDs 2](#_Toc220153590)

[Text S2. Preparation of mixed-salt solution 2](#_Toc220153591)

[Text S3. Selection of the optimal priming concentration of Si-QDs 2](#_Toc220153592)

[Text S4. Statistical analysis 3](#_Toc220153593)

[Table S1. The electric conductivity of NaCl solutions and mixed salt solutions. 4](#_Toc220153594)

[Table S2. The selected properties of the soil used in the present study 4](#_Toc220153595)

[Fig. S1 Effects of different concentrations of Si-QDs on maize germination under stress free conditions 5](#_Toc220153596)

[Fig. S2 Effects of different concentrations of Si-QDs on maize germination under 75 mM mixed-salt stress 6](#_Toc220153597)

[Fig. S3 Effect of seed priming with Si-QDs on maize seed germination under NaCl stress 7](#_Toc220153598)

[Fig. S4 Effect of seed priming with Si-QDs on maize growth. 8](#_Toc220153599)

[Fig. S5 The comprehensive effects of Si-QDs on maize growth. The different small letters reflect a significant difference among the different treatments 8](#_Toc220153600)

[Fig. S6 Effect of nano-priming with Si-QDs on photosynthesis in maize seedlings 8](#_Toc220153601)

[Fig. S7 Effect of seed priming with Si-QDs on Na^+^ and K^+^ content in maize seedlings. 9](#_Toc220153602)

[Fig. S8 Internalization and transport of Si-QDs in maize seeds and seedlings 9](#_Toc220153603)

[References 9](#_Toc220153604)

***Text S1. Preparation of Si-QDs***

Sodium ascorbate (2.3 g; Sinopharm Chemical Reagent Co., Ltd., Shanghai, China) was dissolved in 8.0 mL ultrapure water in a glass beaker. The beaker was placed on a heating magnetic stirrer (IKA, Germany**)** and stirred in a water bath. Once the temperature reached 80 °C, 2.0 mL of *N*-[3-(Trimethoxysilyl)propyl]ethylenediamine (RHAWN, Shanghai, China) was added dropwise, and the mixture was maintained at 80 °C in the water bath for 8 h. During the reaction, the solution color gradually changed from pale yellow to yellow-brown and finally dark brown. After cooling to room temperature, the reaction mixture was transferred into a 1000 Da MWCO dialysis membrane (SpectraPor, USA) and dialyzed against ultrapure water for 48 h, with the dialysate replaced periodically. The retentate was then collected and freeze-dried to obtain Si-QDs powder(Na et al., 2019).

***Text S2. Preparation of mixed-salt solution***

The composition of the mixed-salt solution was designed based on the typical ionic composition of coastal saline-alkali soils in the Yellow River Delta. The ratio was based on the analysis of 193 coastal saline–alkali soil samples (0–30 cm depth) conducted by Fan et al., which reported an ionic molar ratio of Na^+^:Mg^2+^:Ca^2+^:Cl^-^:SO_4_^2-^:HCO_3_^-^ = 15:5:1:17:5:0.1(Fan et al., 2010). According to this molar ratio, a mixed-salt solution of 150 mmol L^-1^ was prepared using NaCl, MgSO_4_, CaCl_2_, and NaHCO_3_ (AR; Sinopharm Chemical Reagent Co., Ltd., China). As an example of preparing 1.0 L of mixed-salt solution, 6.232 g NaCl, 4.279 g MgSO_4_, 0.789 g CaCl_2_, and 0.596 g NaHCO_3_ were accurately weighed with an analytical balance, sequentially dissolved in an appropriate volume of ultrapure water, transferred to a 1 L volumetric flask, brought to the mark, and mixed thoroughly for use.

***Text S3. Selection of the optimal priming concentration of Si-QDs***

Si-QDs powder was dispersed in ultrapure water and sonicated (100 W, 40 kHz, 30 min) to ensure homogeneous dispersion. Si-QDs priming solutions were prepared at concentrations of 0, 100, 200, 500, and 1000 mg L^-1^. Plump and uniform maize seeds were selected and randomly assigned to five groups. Seeds were surface-sterilized in 10% H_2_O_2_ for 30 minutes, thoroughly rinsed with ultrapure water, and then immersed in the Si-QDs solutions at a 1:5 (w/v) seed-to-solution ratio. Priming was conducted in darkness for 24 h in a temperature-controlled orbital shaker (25 °C, 100 rpm; ZQZY-78BV, ZHICHU, China). After priming, seeds were rinsed five times with ultrapure water to remove residual Si-QDs and blotted dry with absorbent paper.

For each priming concentration, two salinity regimes were applied: ultrapure water (control) and 75 mM mixed-salt solution, with six replicates per combination. Primed seeds were placed in Petri dishes (Ø 9 cm) lined with filter paper, with each dish containing 3 mL of ultrapure water or 3 mL of 75 mM mixed-salt solution (5 Si-QDs concentrations × 2 salt regimes × 6 replicates × 10 seeds). Petri dishes were incubated for 7 days at 25 ± 1 °C in darkness in an artificial climate chamber (RXZ-380C, Ningbo Jiangnan Instrument Factory, China). Germination was recorded daily, defining germination as radicle emergence ≥ 2 mm. Germination rate (GR), germination potential (GP), and germination index (GI) were calculated(Sun et al., 2024). After 7 days of cultivation, the shoot and root lengths were measured with a ruler (1 mm minimum division) for all germinated maize seedlings, and the vigor index (VI) was calculated(Yan et al., 2023; Kang et al., 2025).

***Text S4. Statistical analysis***

In the structural equation model (SEM) (Liu et al., 2023) , we established six vectors: “Si-QDs”, “Antioxidant defenses”, “Na^+^/K^+^”, “Photosynthetic performance”, “Plant biomass” and “Plant morphological features”. The vector “Si-QDs” indicated the treatment with Si-QDs seed priming (“0” for CK or H_2_O priming; “1” for Si-QDs priming). All variables besides “Si-QDs” were standardized by log10 transformation to improve normality. A principal component analysis (PCA) was applied to simplify the variables of “Antioxidant defenses”, “Na^+^/K^+^”, “Photosynthetic performance”, “Plant biomass”, and “Plant morphological features” subjected to SEM. Specifically, “Antioxidant defenses” was represented by the PCA axis (PCA1), explaining 84.05% of the variation in SOD activity and MDA content. “Na^+^/K^+^” was represented by the PCA1, explaining 89.06% of the variation in leaf and root Na^+^/K^+^. “Photosynthetic performance” was represented by the PCA1, explaining 63.14% of the variation in net photosynthetic rate, transpiration rate, stomatal conductance, and SPAD. “Plant biomass” was represented by the PCA1, explaining 91.32% of the variation in shoot and root fresh and dry weight. “Plant morphological features” was represented by the PCA1, explaining 78.55% of the variation in plant height, stem diameter, root length, root area, root volume, and root vigor. We sequentially eliminated nonsignificant pathways unless the pathways were biologically informative.

Table S1. The electric conductivity of NaCl solutions and mixed salt solutions (mean ± SD, n = 3).

| Solution concentration (mM) | NaCl (dS m^-1^) | Mixed-salt (dS m^-1^) |
| --- | --- | --- |
| 0 | 0 | 0 |
| 25 | 2.52 ± 0.02 | 2.51 ± 0.01 |
| 50 | 5.09 ± 0.01 | 4.99 ± 0.01 |
| 75 | 7.51 ± 0.01 | 7.50 ± 0.01 |
| 100 | 9.97 ± 0.01 | 9.97 ± 0.01 |
| 125 | 12.39 ± 0.01 | 12.41 ± 0.01 |
| 150 | 14.97 ± 0.02 | 14.99 ± 0.01 |

Table S2. The selected properties of the soil used in the present study (mean ± SD, n = 3).

| Properties | Sediment |
| --- | --- |
| pH | 8.54 ± 0.06 |
| ECe^α^ (dS m^-1^) | 7.32 ± 0.21 |
| TOC^β^ (g kg^-1^) | 1.93 ± 0.05 |
| TN^γ^ (g kg^-1^) | 0.19 ± 0.02 |
| NH_4_^+^-N (mg kg^-1^) | 7.45 ± 0.15 |
| NO_3_^-^-N (mg kg^-1^) | 6.51 ± 0.21 |
| AP^ε^ (mg kg^-1^) | 7.61 ± 0.28 |

^α^ ECe: electrical conductivity of saturation extract.

^β^ TOC: total organic carbon.

^γ^ TN: total nitrogen

^ε^ AP: available phosphorus.


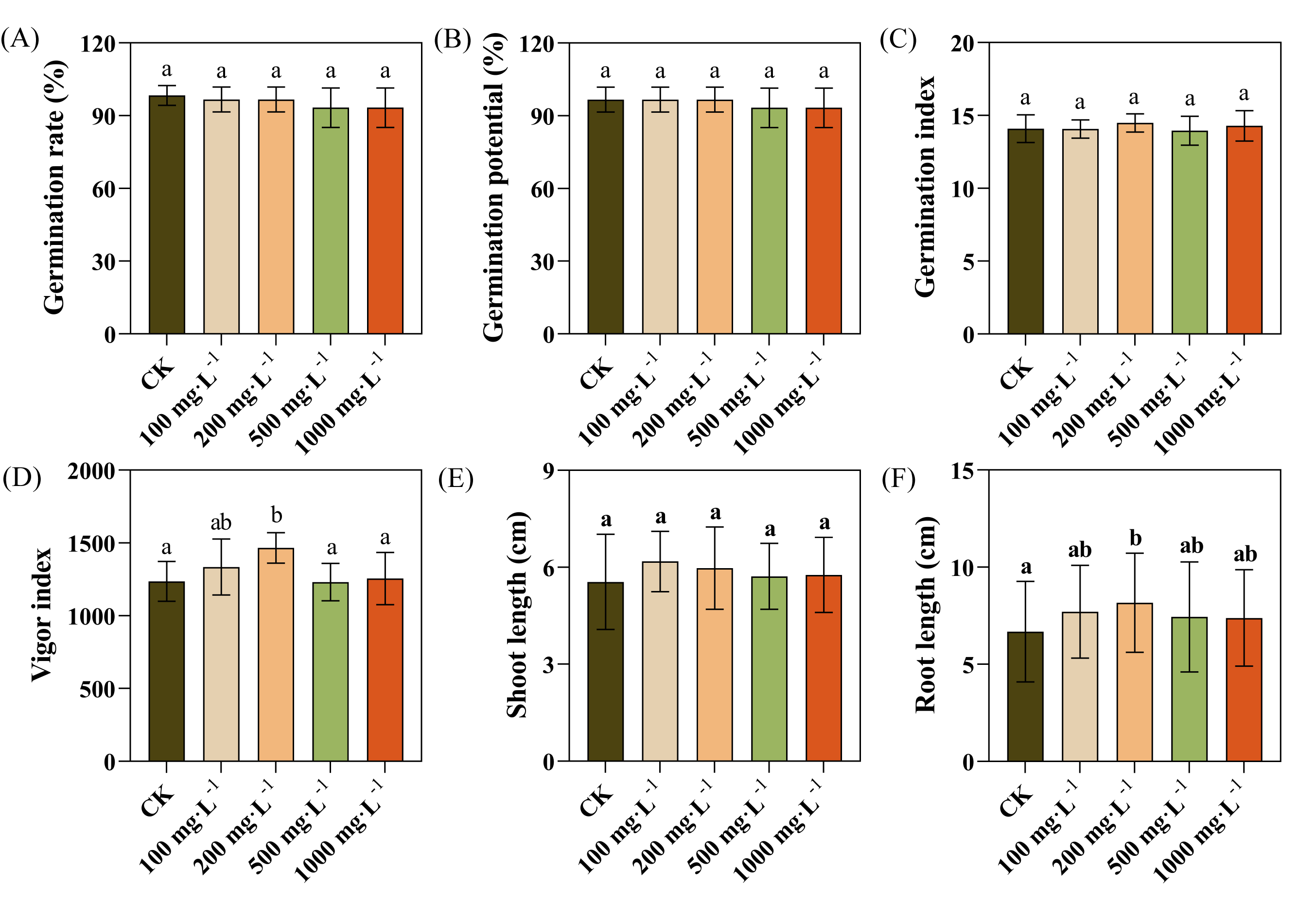


**Figure S1** Effects of different concentrations of Si-QDs on maize germination under stress free conditions. (A) Germination rate, (B) Germination potential, (C) Germination index, (D) Vigor index, (E) Shoot length, (F) Root length. Data are means ± the standard deviation (*n* = 6). The different small letters reflect a significant difference among the different treatments (Duncan’s multiple-comparison test, *P* < 0.05). Under ultrapure-water conditions, none of the Si-QDs priming concentrations differed from CK for germination rate (GR), germination potential (GP), germination index (GI), or 7-day seedling shoot length (*P* > 0.05). By contrast, 200 mg L^-1^ Si-QDs priming solution significantly increased 7-day seedling root length and the vigor index (VI) by 22.28% and 19.07%, respectively (*P* < 0.05).


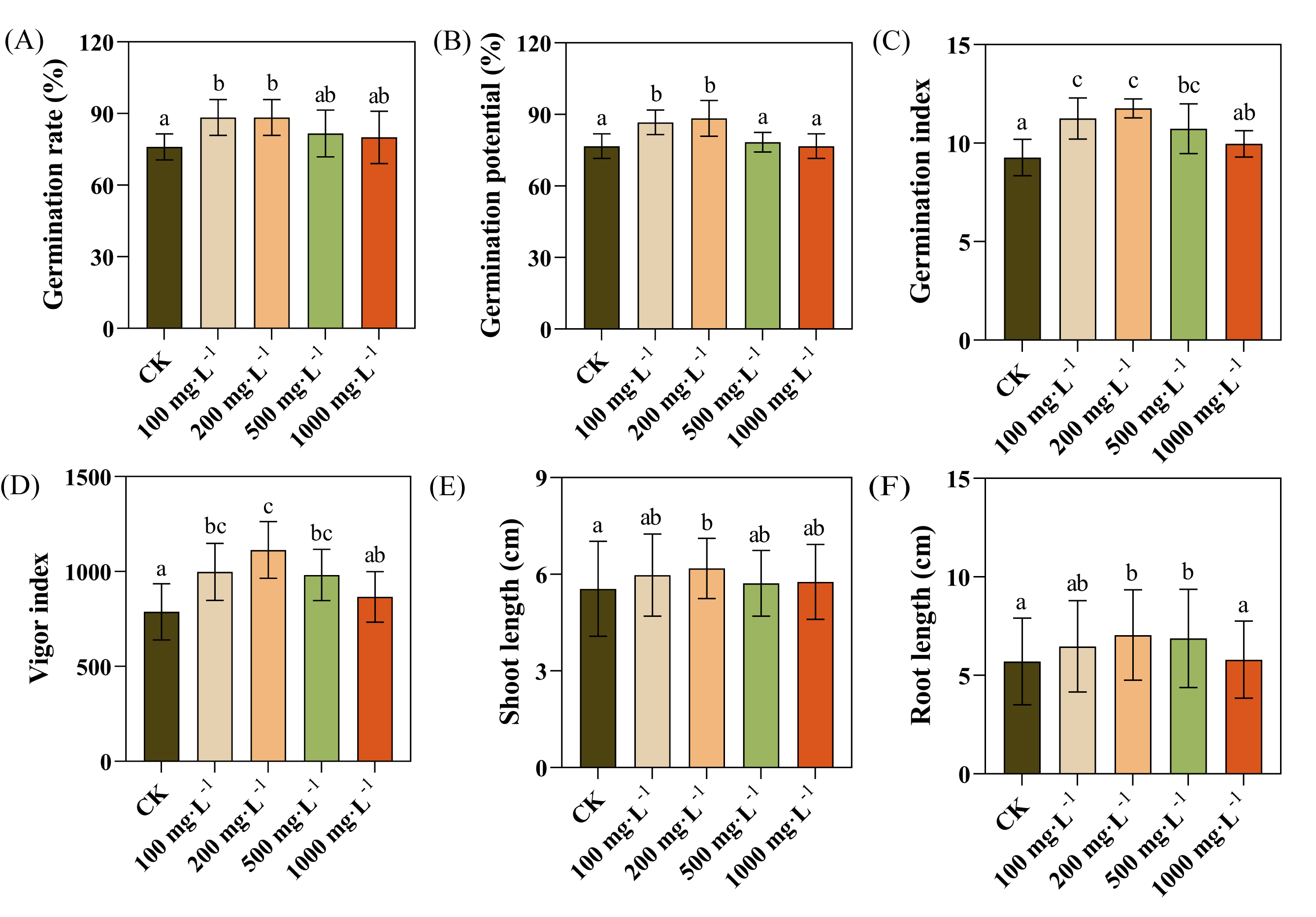


**Figure S2** Effects of different concentrations of Si-QDs on maize germination under 75 mM mixed-salt stress. (A) Germination rate, (B) Germination potential, (C) Germination index, (D) Vigor index, (E) Shoot length, (F) Root length. Data are means ± the standard deviation (*n* = 6). The different small letters reflect a significant difference among the different treatments (Duncan’s multiple-comparison test, *P* < 0.05). Under 75 mM mixed-salt stress, all Si-QDs priming concentrations improved maize seed germination relative to CK. The 200 mg L^-1^ Si-QDs priming solution produced the greatest gains, significantly increasing germination rate (GR), germination potential (GP), germination index (GI), vigor index (VI), and 7-day seedling shoot and root lengths by 16.23%, 15.22%, 26.93%, 41.40%, 23.87%, and 23.52%, respectively (*P* < 0.05), outperforming all other concentrations. Considering both ultrapure-water and 75 mM mixed-salt treatments, 200 mg L^-1^ was selected as the optimal Si-QDs priming concentration.


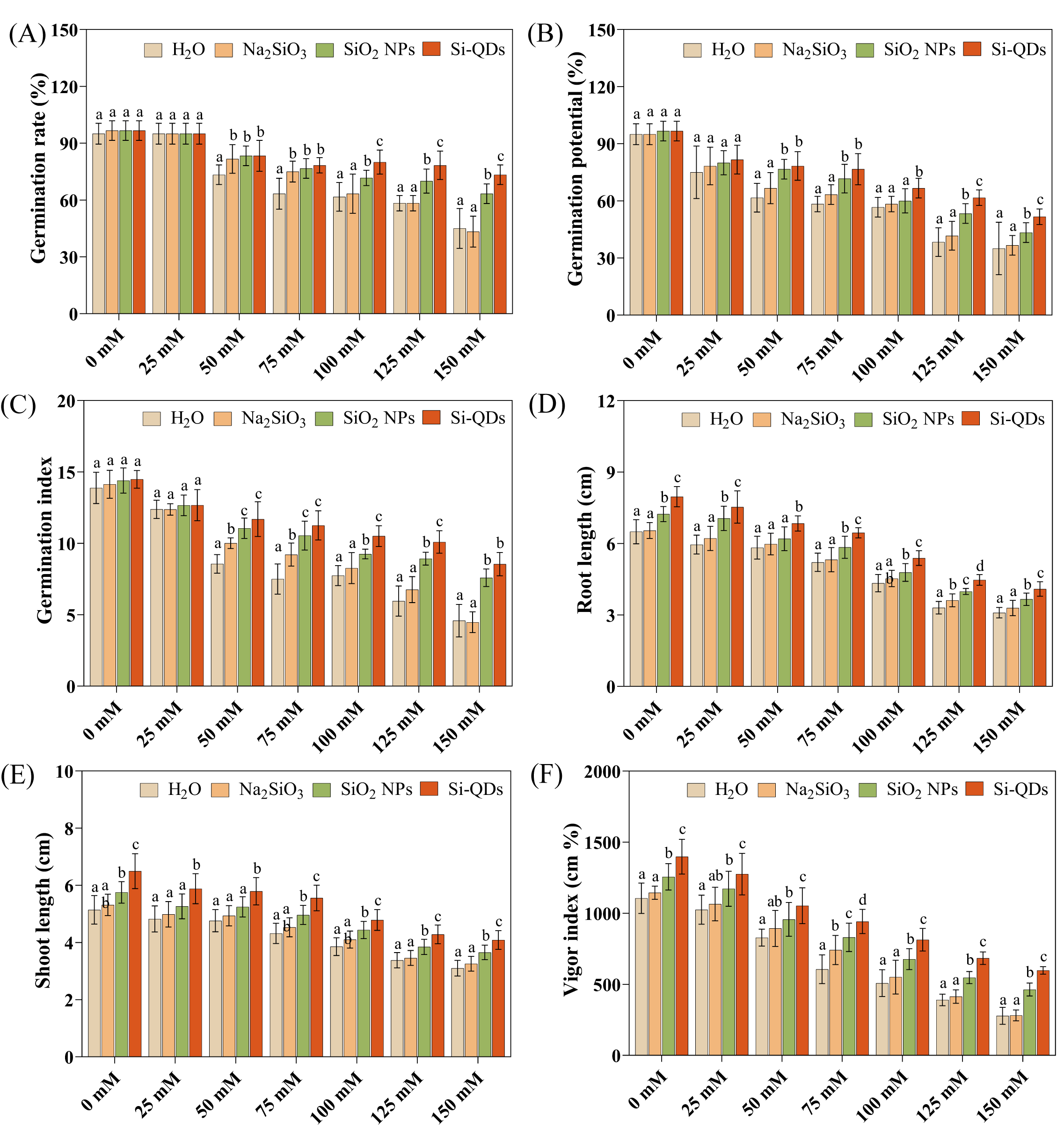


**Figure S3** Effect of seed priming with Si-QDs on maize seed germination under NaCl stress. (A) Germination rate, (B) Germination potential, (C) Germination index, (D) Shoot length, (E) Root length, (F) Vigor index. Data are means ± the standard deviation (*n* = 6). The different small letters reflect a significant difference among the different treatments (Duncan’s multiple-comparison test, *P* < 0.05).

**
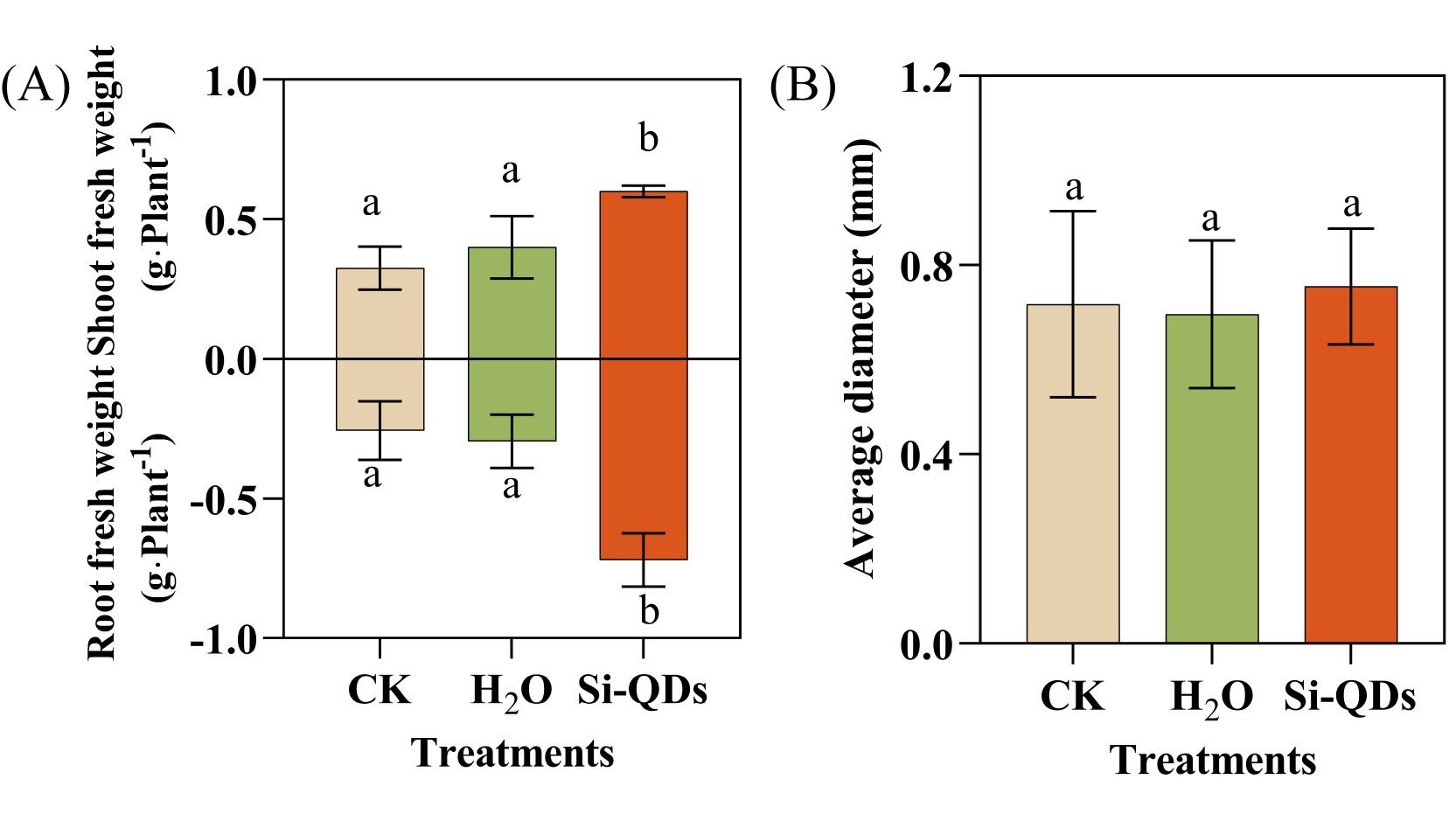
**

**Figure S4** Effect of seed priming with Si-QDs on maize growth. (A) Shoot and root fresh weight, (B) Average diameter. Data are means ± the standard deviation (*n* = 5). The different small letters reflect a significant difference among the different treatments (Duncan’s multiple-comparison test, *P* < 0.05).





**Figure S5** The comprehensive effects of Si-QDs on maize growth. The different small letters reflect a significant difference among the different treatments (Duncan’s multiple-comparison test, *P* < 0.05).


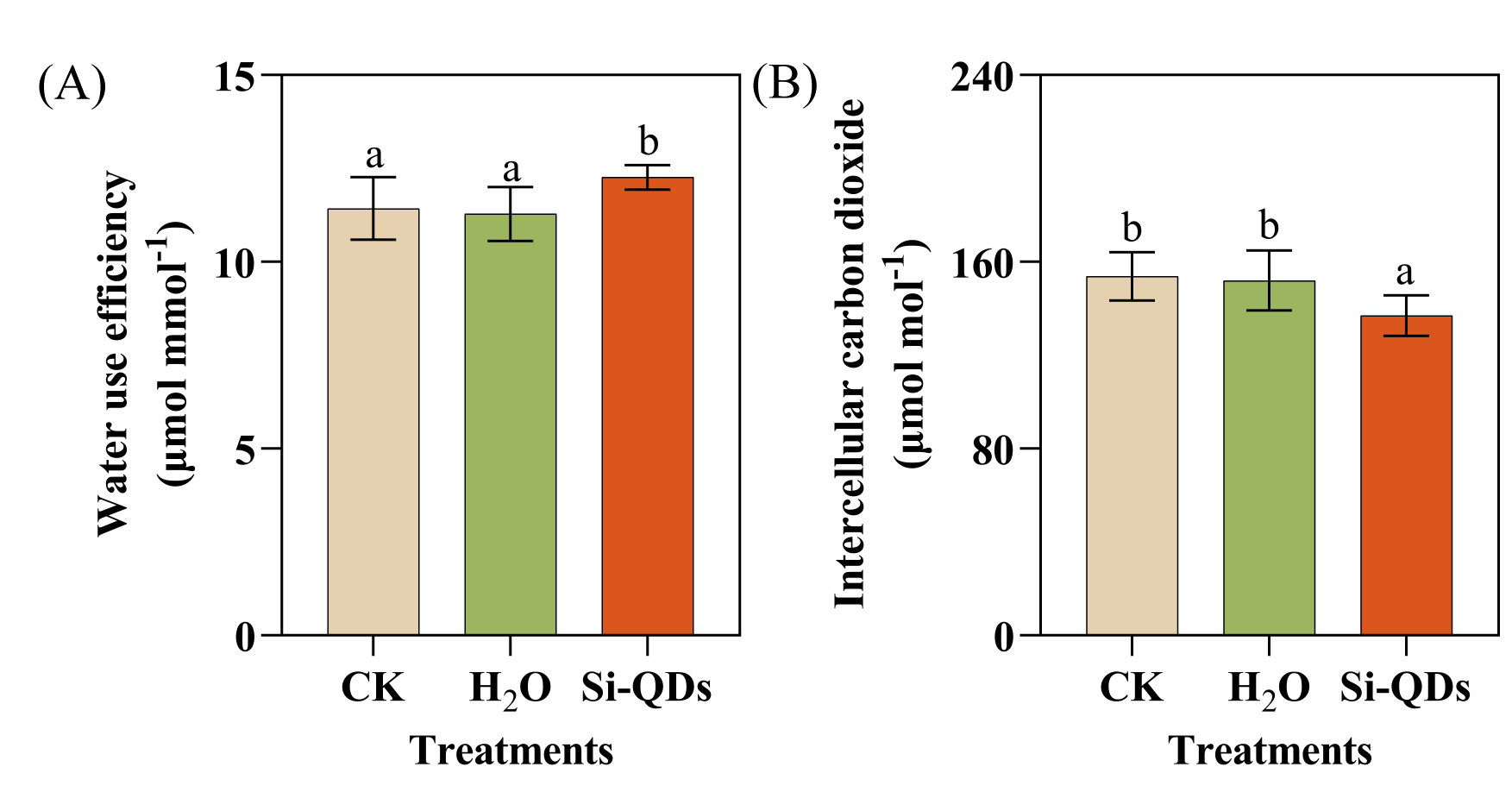


**Figure S6** Effect of nano-priming with Si-QDs on photosynthesis in maize seedlings. (A) Water use efficiency, (B) Intercellular CO_2_ concentration. Data are means ± the standard deviation (*n* = 5). The different small letters reflect a significant difference among the different treatments (Duncan’s multiple-comparison test, *P* < 0.05).

**
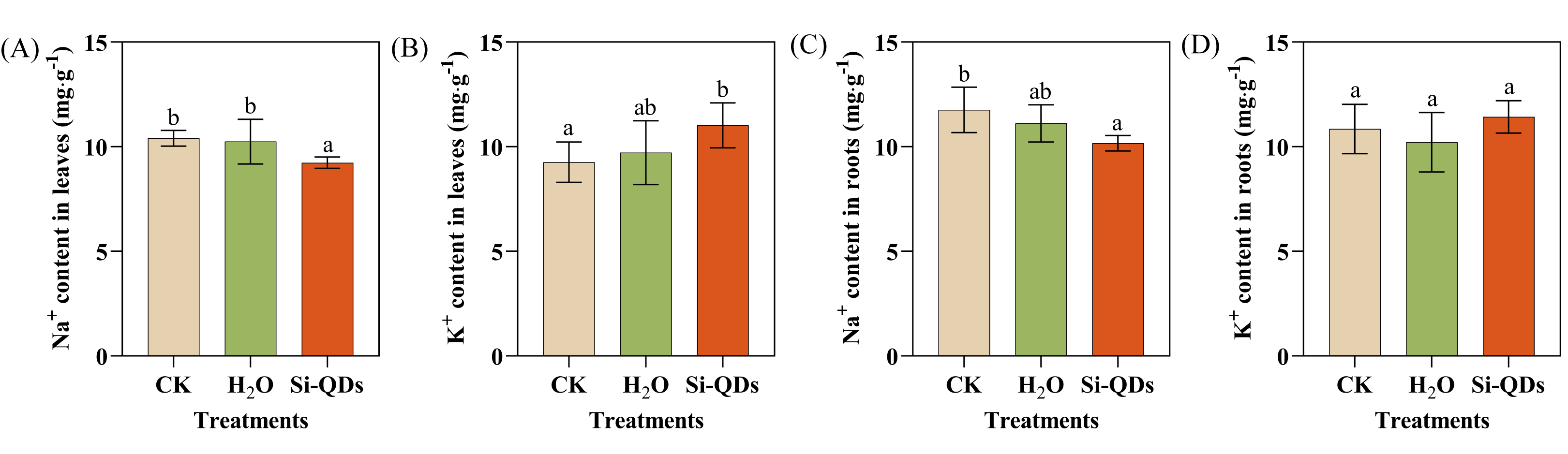
**

**Figure S7** Effect of seed priming with Si-QDs on Na^+^ and K^+^ content in maize seedlings. (A) Na^+^ in leaves, (B) K^+^ in leaves, (C) Na^+^ in roots, (D) K^+^ in roots. Data are means ± the standard deviation (*n* = 5). The different small letters reflect a significant difference among the different treatments (Duncan’s multiple-comparison test, *P* < 0.05).


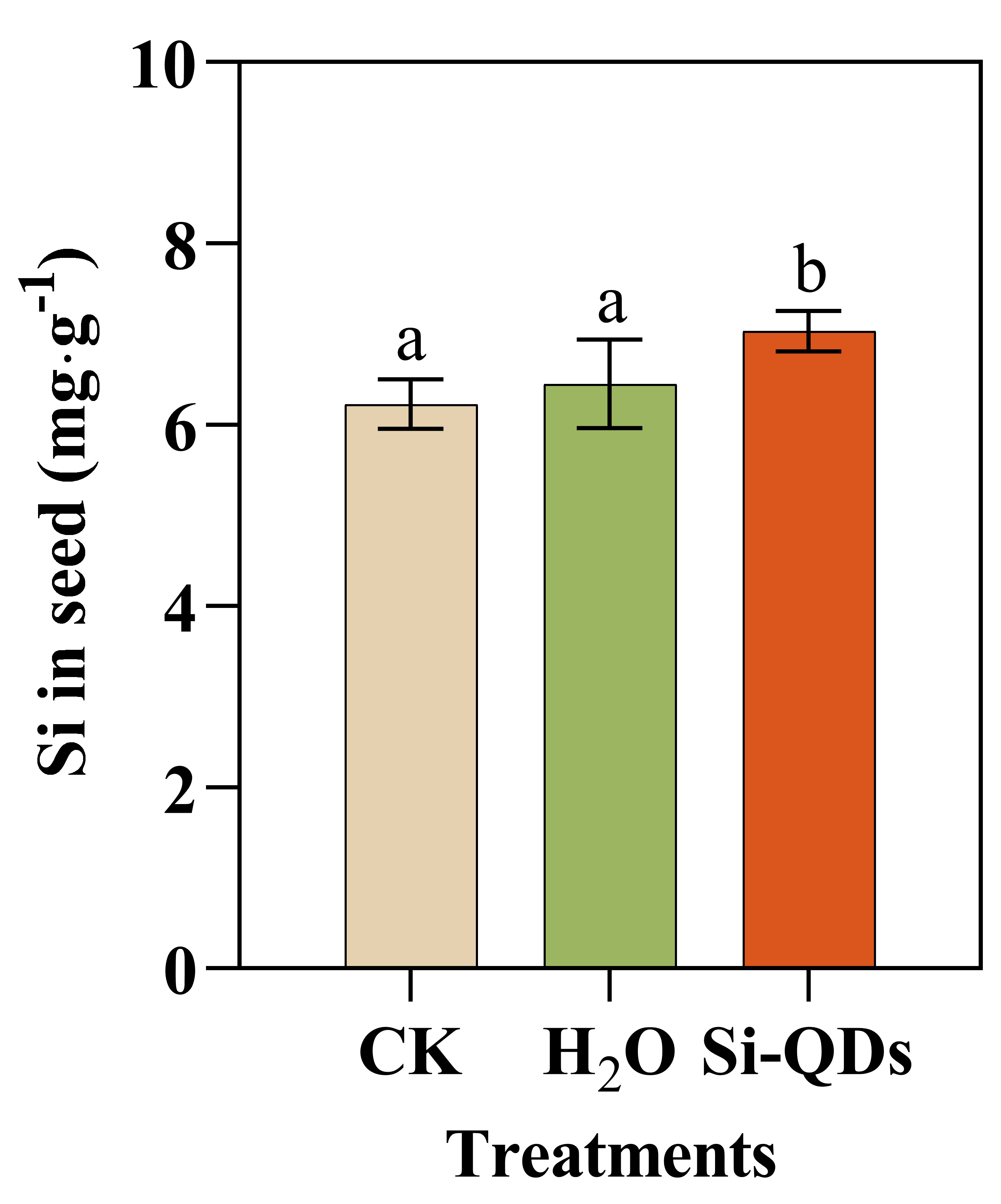


**Figure S8** Internalization and transport of Si-QDs in maize seeds and seedlings. Total silicon content of maize seeds Data are means ± the standard deviation (*n* = 5). The different small letters reflect a significant difference among the different treatments (Duncan’s multiple-comparison test, *P* < 0.05).

**References**

Fan, X., Liu, G., Tang, Z., and Shu, L. (2010). Analysis on Main Contributors Influencing Soil Salinization of Yellow River Delta. *Journal of Soil and Water Conservation* 24(01)**,** 139–144. doi: <https://doi.org/10.13870/j.cnki.stbcxb.2010.01.030>.

Kang, Z., Lu, J., Zheng, S., Hu, X., Wang, L., Jiang, L., et al. (2025). Silica-Activated Redox Signaling Confers Rice with Enhanced Drought Resilience and Grain Yield. *ACS Nano* 19(3)**,** 3752–3763. doi: <https://doi.org/10.1021/acsnano.4c14608>.

Liu, D., Iqbal, S., Gui, H., Xu, J., An, S., and Xing, B. (2023). Nano-Iron Oxide (Fe_3_O_4_) Mitigates the Effects of Microplastics on a Ryegrass Soil–Microbe–Plant System. *ACS Nano* 17(24)**,** 24867–24882. doi: <https://doi.org/10.1021/acsnano.3c05809>.

Na, M., Chen, Y., Han, Y., Ma, S., Liu, J., and Chen, X. (2019). Determination of potassium ferrocyanide in table salt and salted food using a water-soluble fluorescent silicon quantum dots. *Food Chemistry* 288**,** 248–255. doi: <https://doi.org/10.1016/j.foodchem.2019.02.111>.

Sun, S., Peng, L., Huang, Q., Huang, Z., Wang, C., Zhao, J., et al. (2024). Genome-wide association study reveals that *JASMONATE ZIM-DOMAIN 5* regulates seed germination in rice. *The Crop Journal* 12(4)**,** 1001–1009. doi: <https://doi.org/10.1016/j.cj.2024.05.007>.

Yan, X., Chen, S., Pan, Z., Zhao, W., Rui, Y., and Zhao, L. (2023). AgNPs-Triggered Seed Metabolic and Transcriptional Reprogramming Enhanced Rice Salt Tolerance and Blast Resistance. *ACS Nano* 17(1)**,** 492–504. doi: <https://doi.org/10.1021/acsnano.2c09181>.
