# Supplementary figures and images for: Seed priming with silicon quantum dots promotes maize seedling establishment in coastal saline soil
Source: Front Plant Sci. 2026 Apr 14;17:1796045. doi: 10.3389/fpls.2026.1796045 (PMC13120962; doi:10.3389/fpls.2026.1796045)

**Graphical abstract**


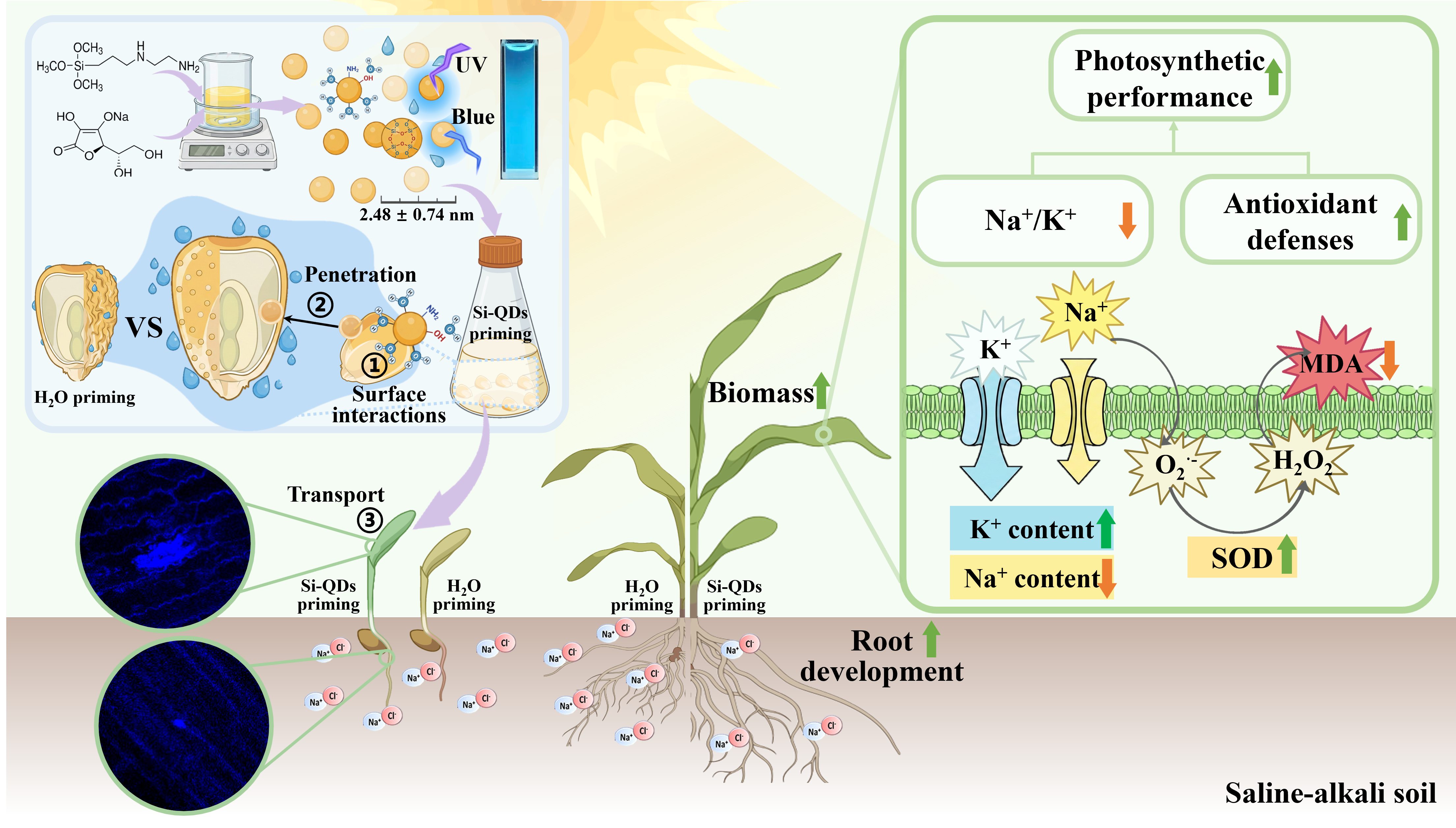

Supplement: Supplementary file 2 [file SupplementaryFile2.docx]
